# Genetic Deletion of the Purinergic Receptor *P2rx7* Worsens the Phenotype of α‑Sarcoglycan Muscular Dystrophy

**DOI:** 10.1021/acsptsci.5c00138

**Published:** 2025-09-11

**Authors:** Cecilia Astigiano, Elisa Principi, Sara Pintus, Andrea Benzi, Serena Baratto, Chiara Panicucci, Mario Passalacqua, Juan Sierra-Marquez, Annette Nicke, Francesca Antonini, Genny Del Zotto, Annunziata Gaetana Cicatiello, Lizzia Raffaghello, Tanja Rezzonico Jost, Fabio Grassi, Santina Bruzzone, Claudio Bruno, Elisabetta Gazzerro

**Affiliations:** † Department of Experimental Medicine, Section of Biochemistry, 9302 University of Genoa, 16132 Genoa, Italy; ‡ Center of Translational and Experimental Myology, IRCCS Istituto G. Gaslini, 16147 Genoa, Italy; § Laboratory of Gene Expression Regulation, 9246 IRCCS Ospedale Policlinico San Martino, 16132 Genoa, Italy; ∥ Walther Straub Institute of Pharmacology and Toxicology, Faculty of Medicine, LMU Munich, 80336 Munich, Germany; ⊥ Core facilities Department of Research and Diagnostics, IRCCS Istituto G. Gaslini, 16147 Genoa, Italy; # Department of Clinical Medicine and Surgery, University of Naples “Federico II”, 80138 Naples, Italy; ∇ Molecular Oncology and Angiogenesis Unit, 9246 IRCCS Ospedale Policlinico San Martino, 16132 Genoa, Italy; ○ Institute of Oncology Research (IOR), 6500 Bellinzona, Switzerland; ◆ Istituto Nazionale Genetica Molecolare ‘‘Romeo ed Enrica Invernizzi’’, 20122 Milan, Italy; ¶ Department of Medical Biotechnology and Translational Medicine, University of Milan, 20133 Milan, Italy; & 9246 IRCCS Ospedale Policlinico San Martino, 16132 Genoa, Italy; ● Department of Neurosciences, Rehabilitation, Ophthalmology, Genetics, Maternal and Child Health (DINOGMI), 9302 University of Genoa, 16132 Genoa, Italy; ◊ Unit of Muscle Research, Experimental and Clinical Research Center, Charité Universitätsmedizin and Max Delbrück Research Center for Molecular Medicine, 10627 Berlin, Germany

**Keywords:** α-sarcoglicanopathy, LGMDR3, P2X7R, skeletal muscle, fibrosis, inflammation

## Abstract

Limb-girdle muscular dystrophy R3 (LGMDR3), a rare genetic disorder characterized by progressive impairment of limb, diaphragmatic, and respiratory muscles, is caused by loss-of-function mutations in the α-sarcoglycan gene (*SGCA*) and aggravated by immune-mediated damage and fibrotic tissue replacement. Pharmacological inhibition of purinergic receptor P2X7 (P2X7R) reduced inflammation and fibrosis in *Sgca* ^–/–^ mice. To further define the role of P2X7R, we generated a double knockout mouse model *Sgca* ^*–/–*^ *P2rx7* ^*‑/*‑^. We compared diaphragms isolated from 24-week-old *Sgca* ^–/–^ *P2rx7* ^+/+^ and *Sgca* ^*–/–*^ *P2rx7*^–/–^** mice since the diaphragmatic muscle is early and severely damaged by *Sgca* genetic loss-of-function. Unexpectedly, *Sgca* ^*–/–*^ *P2rx7^–/–^* mice displayed increased extracellular matrix deposition and augmented cellularity in fibrotic areas, in particular, a higher number of CD3^+^ lymphocytes and Iba1^+^ macrophages compared to *Sgca* ^–/–^ *P2rx7* ^*+/+*^ mice. Moreover, intense P2X4R signal colocalized with CD3^+^ and Iba1^+^ cells, confirming its expression by these infiltrating immune cells. Absence of an improvement of the dystrophic phenotype was histologically confirmed in *Sgca* ^*–/–*^ *P2rx7^–/–^* quadriceps, although the fibrotic reaction was milder than that in diaphragms, suggesting a differential influence of the tissue microenvironment on the receptor functions. Flow cytometric analysis of limb muscle-infiltrating immune cells revealed a decrease in NK cells. Motor performance tests did not reveal any difference between the two genotypes. In conclusion, this study identified a divergent outcome of genetic deletion of the *P2rx7* gene as compared to P2X7R blockade in α-sarcoglycan dystrophic tissue, suggesting that pharmacological interventions targeting the P2X7R in dystrophic immune-mediated damage require careful definition of a precise time window and dosage.

## Graphical abstract

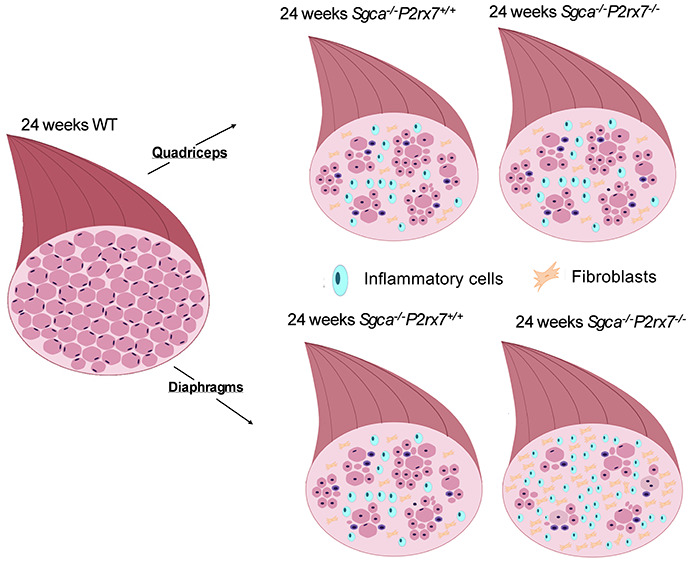

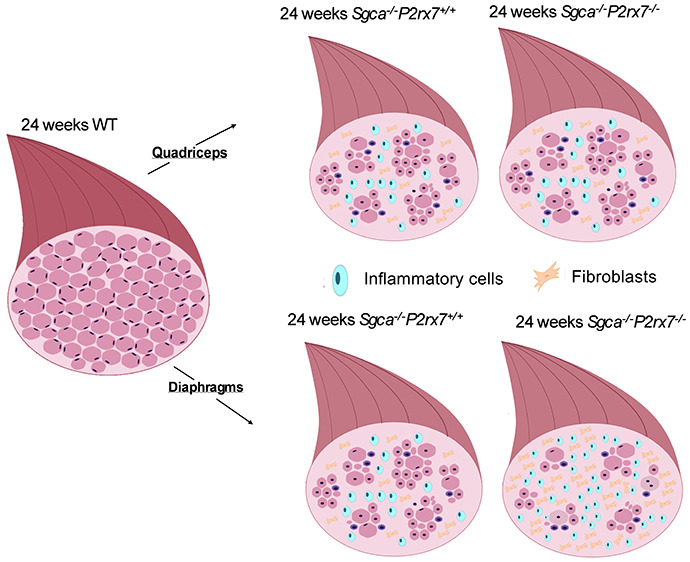

Limb girdle muscular dystrophy (LGMD) is a heterogeneous group of genetic muscular disorders primarily characterized by muscular weakness of the scapular and pelvic girdles.^1^

Sarcoglycanopathies, the most severe forms of recessive LGMDs, accounting for 10–25% of all cases, are due to mutations in the sarcoglycan genes, *SGCA, SGCB, SGCD*, and *SGCG*, encoding for the α-, β-, γ-, and δ-sarcoglycan transmembrane glycoproteins, and are classified as LGMDR3, LGMDR4, LGMDR5, and LGMDR6, respectively.^2,3^

The four sarcoglycans are components of the dystrophin-associated protein complex (DAPC), which is expressed in the sarcolemma of skeletal and cardiac muscle and provides structural stability to the plasma membrane during muscle contraction. When one protein member of the complex is not expressed because of a primary genetic defect, the others can be secondarily reduced as a result of mis-localization and enhanced proteasomal degradation.^4^ Genetic alterations in sarcoglycan or dystrophin encoding genes determine myofiber degeneration and necrosis, inflammation and fibrosis, although with a variable spectrum of severity.^1,5,6^

Interestingly, next to its structural function, the α-sarcoglycan protein (α-SG) exhibits an ATPase activity, which accounts for approximately 25% of the total extracellular ATP hydrolysis.^7^ Indeed, in muscle, eATP can act as a damage-associated molecular pattern (DAMP), triggering the inflammation response, which facilitates the clearance of damaged tissues and prompts regenerative processes, aiming to restore normal muscle function.^8^ However, chronically high concentrations of eATP, as in muscular dystrophies, can activate the ionotropic purinergic receptor P2X7 (P2X7R), which is expressed on immune cells, further exacerbating the inflammatory reaction in dystrophic tissue and initiating a feed-forward detrimental loop.^9^

Overexpression of P2X7R protein levels was detected in muscles of *mdx* mice (the most widely used mouse model of Duchenne Muscular Dystrophy (DMD)), and in muscle biopsies of patients affected by DMD and Becker Muscular Dystrophy (BMD), a milder allelic variant of DMD.^10,11^ Higher expression of P2X7R was also identified through transcriptome analysis in *mdx* primary myoblasts.^15^ Accordingly, the administration of Coomassie Brilliant Blue G (a nonspecific P2X7R antagonist) or Zidovudine (a commonly used anti-HIV drug that acts as a P2X7R antagonist) was able to reduce the number of degeneration–regeneration cycles in *mdx* mice.^10,12^ In a further study, the genetic ablation of *P2rx7* in the *mdx* model led to an improvement of a few histological markers of muscular dystrophy and increased expression levels of myogenin, a marker of regenerating myoblasts. Moreover, the absence of P2X7R hampered inflammation, reduced fibrosis, and the number of infiltrating macrophages, while promoting immunosuppressive regulatory T cell function.^13^

In addition to P2X7R, P2X4R has been reported to be overexpressed in muscle-infiltrating immune cells in *mdx* mice.^10,14^ Nevertheless, transcriptome analysis revealed a higher expression of P2X4R on isolated myoblasts from *mdx* mice.^15^ P2X4R up-regulation at protein and mRNA levels was confirmed also in muscular biopsies of patients affected by DMD or BMD.^11^ In isolated muscles of *Sgca* ^*–/–*^ mice (the mouse model for LGMDR3), in agreement with the studies in *mdx* mice, immunofluorescence staining demonstrated that the P2X4R is not expressed by muscle cells but colocalizes with CD45, a marker for immune cells.^16^

In light of these data, we hypothesized that the tissue microenvironment of dystrophin or α-SG-deficient muscle could be highly rich in eATP, being released by necrotic myofibers and thus able to accumulate and activate purinergic receptors because of the absence of α-SG ATPase activity.

To test whether targeting P2X7R and P2X4R could represent a promising strategy to treat these muscle disorders, we previously analyzed the effects of P2XR7 antagonists. Inhibition of P2X receptors with systemically administered oxidized ATP (oATP), a broad-spectrum P2XR antagonist, in *mdx* and *Sgca* ^*–/–*^ mice enhanced muscle strength and improved muscular structure, while also reducing necrosis. This effect was accompanied by a decrease in immune cell infiltration and an increase in regulatory T cells, which contribute positively to muscle regeneration.^11,16^ To prove the specific involvement of P2X7R, we also used the selective P2X7R antagonist A438079 in the *Sgca* ^*–/–*^ model. In support of our hypothesis, A438079 improved motor function, decreased serum creatine kinase (CK) levels (a systemic marker of muscle tissue damage), and ameliorated the fibrotic and inflammatory reaction.^17^

Currently, different strategies are investigated to treat muscle degeneration in sarcoglycanopathies, including cell therapy, anti-inflammatory treatments, gene therapy, and gene editing.^18–20^ To further strengthen the rationale for inhibiting P2X7R in LGMDR3, we now aimed to define P2X7R-specific localization in healthy and dystrophic skeletal muscle and analyze the effect of its genetic ablation in *Sgca* ^*–/–*^ mice.

## 2 Results and Discussion

### 2.1 Evaluation of P2X7R Localization in Murine Dystrophic Muscles and in Patients Affected by LGMDR3

To further validate P2X7R as a drug target, we addressed the following two basic questions: (1) the cell type-specific localization of the receptor in myofibers and muscle immune infiltrating cells and (2) the consequences of genetic ablation of the receptor in *Sgca* ^*–/–*^ dystrophic mice.

We analyzed P2X7R expression in muscle cells and infiltrating immune cells by immunofluorescence staining of quadriceps tissue isolated from 24-week-old wild type (WT), *P2rx7* ^*–/–*^, *Sgca* ^*–/–*^ *P2rx7* ^*+/+*^, and double Knockout *Sgca* ^*–/–*^ *P2rx7* ^*–/–*^.

In the *Sgca* ^*–/–*^ *P2rx7* ^*+/+*^ mice P2X7R colocalized with CD3^+^ (global marker for T lymphocytes) and Iba1^+^ (marker for macrophages) (Figures 1 and S1) and with CD45^+^ (Supplementary Figure 2). P2X7R protein was, however, not detected in WT as well as dystrophic myofibers (Figures 1, S1, and S2). The IgG control is shown in Supplementary Figure 1, whereas, as a positive control, Figure 1D shows a clear P2X7R^+^ staining in a nerve of the diaphragm of *Sgca* ^*–/–*^ *P2rx7* ^*+/+*^ mice.^21,22^

**Figure 1:**
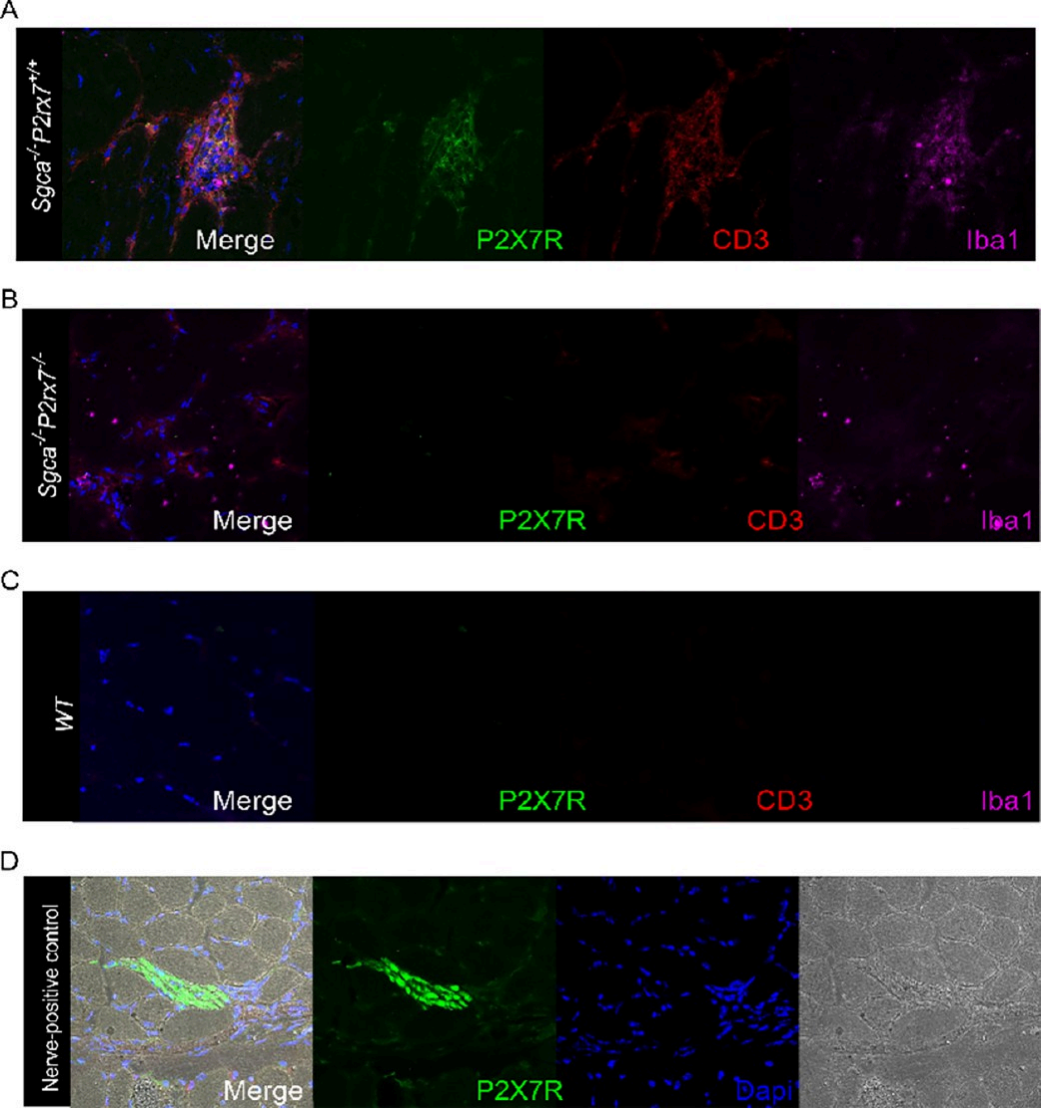
Evaluation of P2X7R expression in biopsies of dystrophic muscles of *Sgca* ^*–/–*^ mice. Representative image of immunofluorescence staining to localize P2X7R, CD3 and Iba1 in skeletal muscle (quadriceps) from *Sgca* ^*–/–*^ *P2rx7* ^*+/+*^ (A) *Sgca* ^*–/–*^ *P2rx7* ^–/–^ (B) and WT (C) mice. (D) Positive control. *n* = 3 images were acquired from 2 slices obtained from *n* = 10 animals.

Although not representing the fully differentiated myofibers, myoblasts were isolated from 3-week-old WT and *Sgca* ^*–/–*^ *P2rx7* ^*+/+*^ puppies. FACS analyses indicated that only 2 and 1.6% of CD56^+^ myoblastic cells,^23^ isolated from WT and *Sgca* ^*–/–*^ *P2rx7* ^*+/+*^, respectively, express the P2X7R (Supplementary Figure 3, panel B, C); a P2X7R^+^ cell line (murine BV2) was used in parallel, as a positive control for the anti-P2X7R antibody (Supplementary Figure 3, panel A).

Taking advantage of the *Tg:Pax7-nGFP* mouse model,^24^ we isolated the tibialis anterior muscle: immunofluorescent analysis using antibodies against GFP (for the Pax7 cells expression) and P2X7R suggested that P2X7R is not expressed in the satellite cells (Supplementary Figure 4). In accordance with the known low number of quiescent Pax7 cells in adult muscle tissue at steady state (i.e, without induced muscle damage), a total of 10 GFP^+^ cells were identified in *n* = 6 acquired images, and none of these cells was positive for the P2X7R staining.^25^

Human P2X7R localization was evaluated in five muscle biopsies obtained from the quadriceps of one healthy control and four genetically confirmed LGMDR3 patients (Supplementary Table 1). Among the LGMDR3 patients, two exhibited mild dystrophic changes with low levels of inflammatory infiltrates, whereas the other two presented severe dystrophic abnormalities, including extensive inflammatory infiltrates and fibrotic areas (Figure 2A). Notably, immunofluorescence analysis revealed P2X7R expression exclusively in the LGMDR3 patients with inflammatory infiltrates (Figures 2D–F and S5), as indicated by the colocalization of the P2X7R signal with CD45, a pan-inflammatory marker for immune cells. Conversely, no expression was detected in the healthy control or in the mildly affected muscle tissue (Figure 2B). As a positive control, Figure 2G shows a clear P2X7R^+^ staining, which colocalizes with Myelin Basic Protein in the myelin sheath of a nerve.

**Figure 2:**
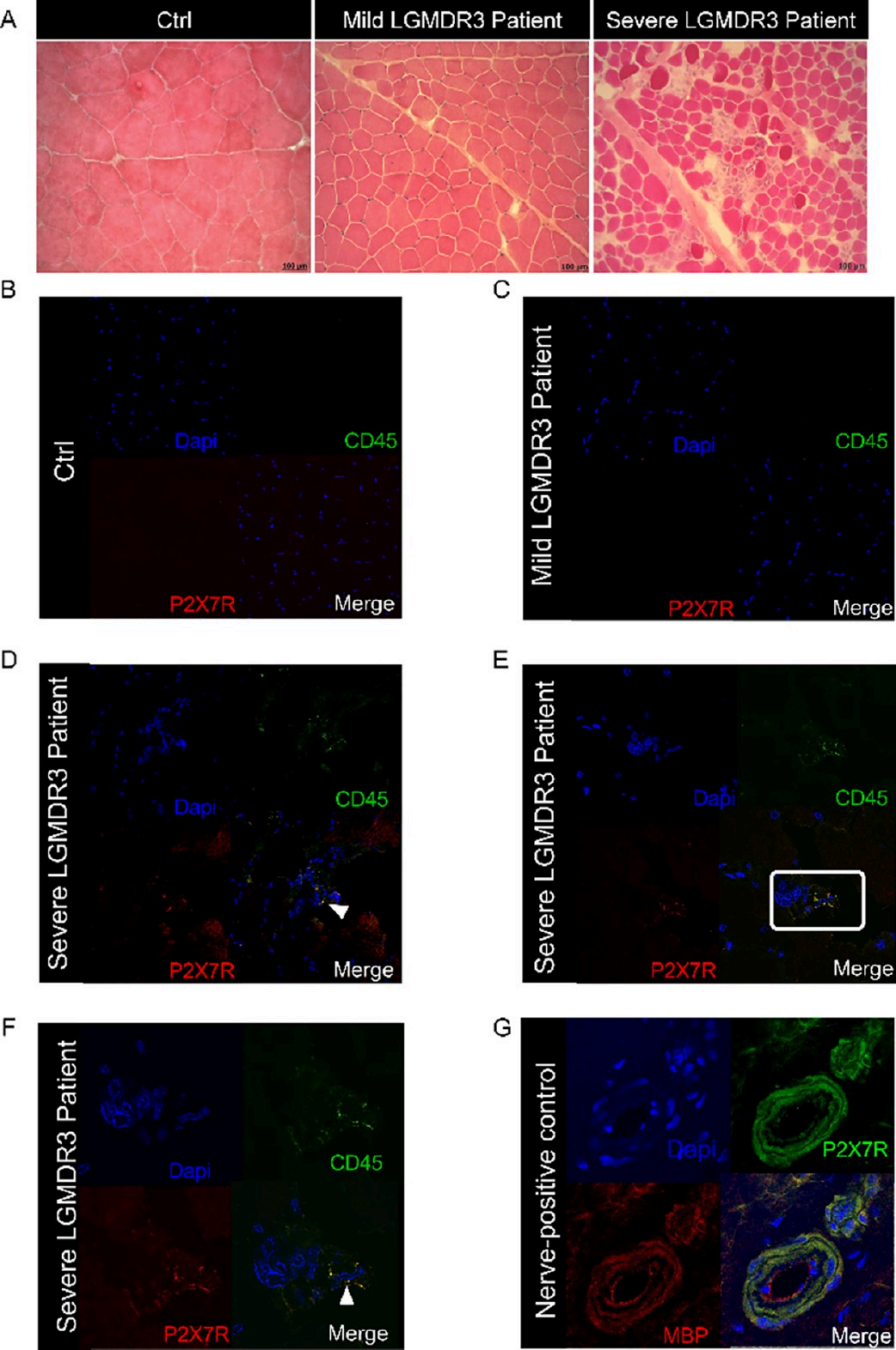
Evaluation of P2X7R expression in biopsies of skeletal muscle from patients affected by LGMDR3. Muscular sections of biopsies from control subjects and patients affected by a mild or a severe form of LGMDR3 were stained with (A) hematoxilin and eosin and (B–F) antibodies against P2X7R and CD45. Representative images are shown. Panel F shows the magnification of the indicated area in panel E. (G) Positive control. Arrows indicate colocalization of CD45 and P2X7R. *n* = 2 images were acquired from one slice from *n* = 4 patients.

Thus, P2X7R was identified neither in healthy nor in dystrophic skeletal muscle cells, despite the previously reported presence of P2X7 mRNA in muscle cells, suggesting that the increased amount of P2X7R protein in mouse and human dystrophic tissue is most likely due to infiltrating cells of innate and adaptive immunity.

We and others had previously highlighted the overexpression of P2X7R transcript and/or increased protein levels in bulk muscle tissue lysates isolated from murine models, from human muscle biopsies, and from human and murine primary or immortalized myoblasts of different muscular dystrophies.^11,14,16,26^ However, the cell type-specific localization of P2X7R remained controversial. Here, we show for the first time immunofluorescence data obtained from biopsies of genetically confirmed LGMDR3 patients. These data are in line with our previous observations in human primary myoblasts isolated from the muscle biopsy of four LGMDR3 patients, where we showed that α-SG-defective cells displayed an increased susceptibility to ATP stimulation with abnormal increase of the intracellular Ca^2+^ ions and enhanced peripheral blood mononuclear cell-chemoattractive properties.^27^ However, P2X7R did not play a prominent role in the ATP-induced toxic and proinflammatory response since neither the use of a P2X7R antagonist nor the absence of extracellular Ca^2+^ reduced the ATP-induced effects. Instead, our data pointed to P2Y2R as the receptor involved in the response to ATP in patient myoblasts.

P2X7R has gathered a lot of interest in recent years as a potential therapeutic target for autoimmune diseases.^28^ However, the results in different experimental models have been inconsistent and sometimes contradictory, and clinical trials using P2X7R inhibitors have reached only phase II for the treatment of rheumatoid arthritis.^29^ Muscular dystrophies, such as dystrophinopathies and sarcoglycanopathies, are not pathologically classified as autoimmune disorders since the primary event is a structural genetic defect. However, an innate inflammatory response and later an adaptive immune-mediated damage as a consequence of released intracellular antigens are a common denominator of the disease trajectory.^5,30^

### 2.2 Absence of P2X7R Aggravates Fibrosis and Inflammation in *Sgca^–/–^* Mice

Since fibrosis is the histopathological hallmark of end-stage muscular dystrophies,^31^ we completed Masson’s trichrome staining in diaphragms and quadriceps of *Sgca* ^*^–/–^*^ *P2rx7* ^*+/+*^ and *Sgca* ^*–/–*^ *P2rx7* ^*–/–*^ mice and analyzed the fractions of fibrotic areas. *Sgca* ^*–/–*^ *P2rx7* ^*+/+*^quadriceps displayed abundant extracellular matrix deposits, which were not significantly modified by *P2rx7* gene deletion (Figure 3A, B, E). Surprisingly, in diaphragms, the most severely damaged muscles by *Sgca* genetic inactivation,^31^ we also observed a significant increase in extracellular matrix deposition in *Sgca* ^*–/–*^ *P2rx7* ^*–/–*^ mice, compared to *Sgca* ^*–/–*^ *P2rx7* ^*+/+*^ mice (Figure 4A, B, E).

**Figure 3:**
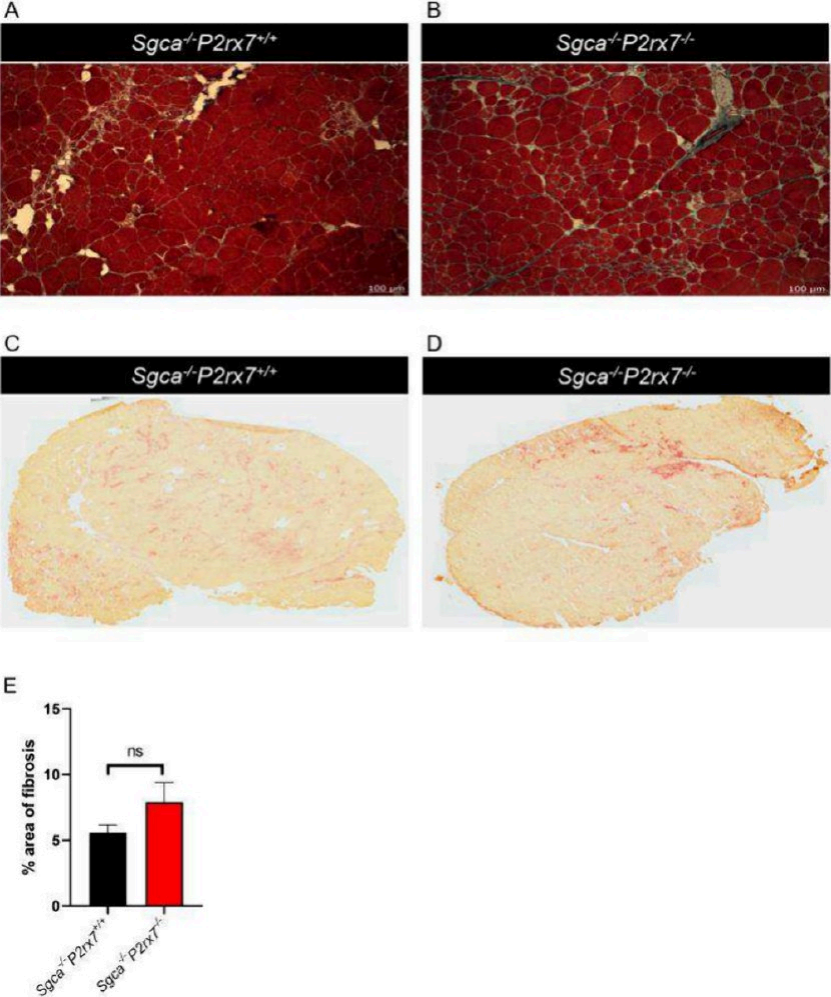
Evaluation of fibrosis and inflammation in quadriceps. Muscular sections of quadriceps from *Sgca* ^*–/–*^ *P2rx7* ^*+/+*^ and *Sgca* ^*–/–*^ *P2rx7* ^*–/–*^ were stained (A, B) with Masson’s trichrome, to reveal the extent of extracellular matrix deposition; (C, D) for acid phosphatase, to evaluate the rate of inflammation. Representative images are shown, and quantification of fibrotic area is reported in panel E. *n* = 2 images were acquired from *n* = 10 animals; ns, not statistically different.

**Figure 4:**
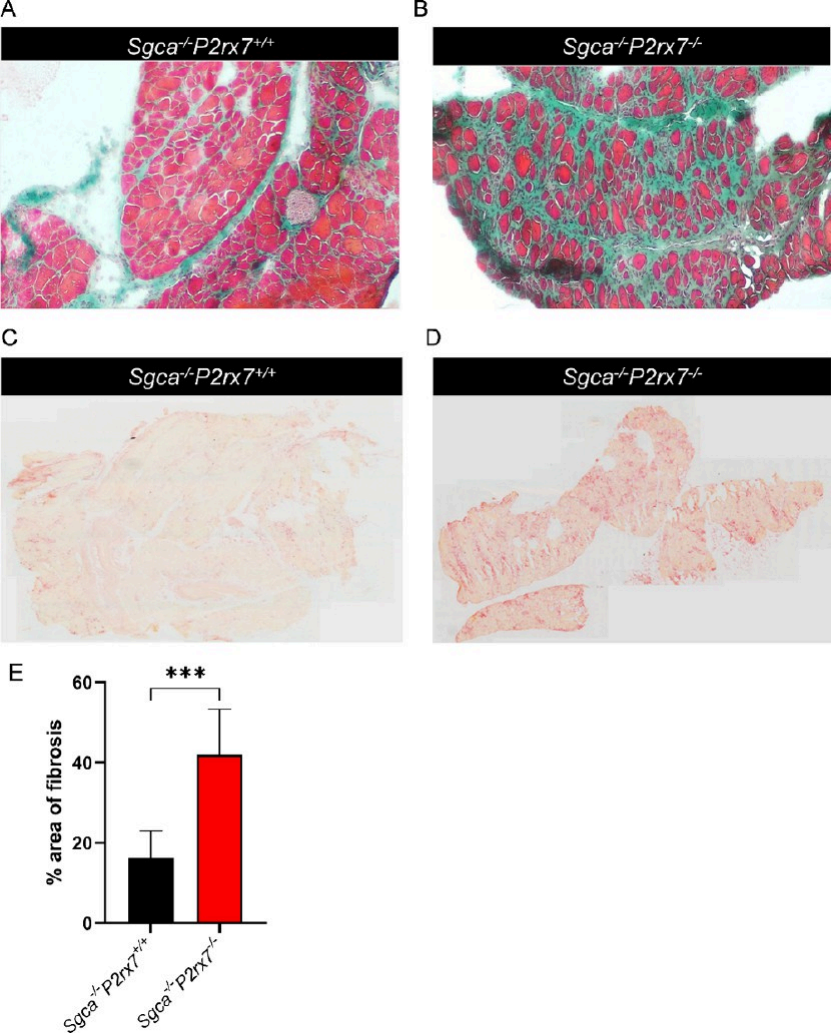
Evaluation of fibrosis and inflammation in diaphragms. Muscular sections of quadriceps from *Sgca* ^*–/–*^ *P2rx7* ^*+/+*^ and *Sgca* ^*–/–*^ *P2rx7* ^*–/–*^ were stained (A, B) with Masson’s trichrome, to reveal the extent of extracellular matrix deposition; (C, D) for acid phosphatase, to evaluate the rate of inflammation. Representative images are shown and quantification of fibrotic area is reported in panel E. *n* = 2 images were acquired from *n* = 10 animals; *, *p* < 0.001.

To investigate the role of P2X7R in inflammation, quadriceps and diaphragm sections from *Sgca* ^*–/–*^ *P2rx7* ^*+/+*^ and *Sgca* ^*–/–*^ *P2rx7* ^*–/–*^ mice were stained with acid phosphatase, which provides a red positive signal in activated inflammatory cells and degenerative myofibers. In accordance with the previous results, the acid phosphatase-positive area from the two mouse strains was not significantly different in the quadriceps (Figure 3C, D) and was enhanced in the diaphragms of *Sgca* ^*–/–*^ *P2rx7* ^*–/–*^ mice (Figure 4C, D).

Next, we performed a cytometric analysis on a pool of limb muscles, including gastrocnemius, quadriceps, and anterior tibialis, isolated from *Sgca* ^*–/–*^ *P2rx7* ^*+/+*^ and *Sgca* ^*–/–*^ *P2rx7* ^*–/–*^ mice. In both genotypes, we identified hematopoietic immune cells. In *Sgca* ^*–/–*^ *P2rx7* ^*–/–*^, as compared to *Sgca* ^*–/–*^ *P2rx7* ^*+/+*^ mice, a reduction was registered in two populations of muscle-infiltrating immune cells, natural killer (NK) and neutrophils. The other identified immune cell populations (lymphocytes, macrophages, monocytes, and dendritic cells) were not different between the two mouse strains (Figure 5A). The reduced infiltration of NK and neutrophils in *Sgca* ^*–/–*^ *P2rx7* ^*–/–*^ mice suggests that P2X7R-mediated migration may be particularly relevant for these two cell populations. Indeed, the role for P2X7R in neutrophil migration has been demonstrated in different sites of inflammation: this role can be direct, i.e., dependent on the P2X7R expressed on neutrophils,^32,33^ or it can also be indirect, i.e., dependent on neutrophil-attracting factors released by other cell types upon P2X7R stimulation. Specifically, Kawamura et al. demonstrated that the ATP-induced P2X7R activation on macrophages determines the release of molecules, inducing neutrophil migration.^34^

**Figure 5:**
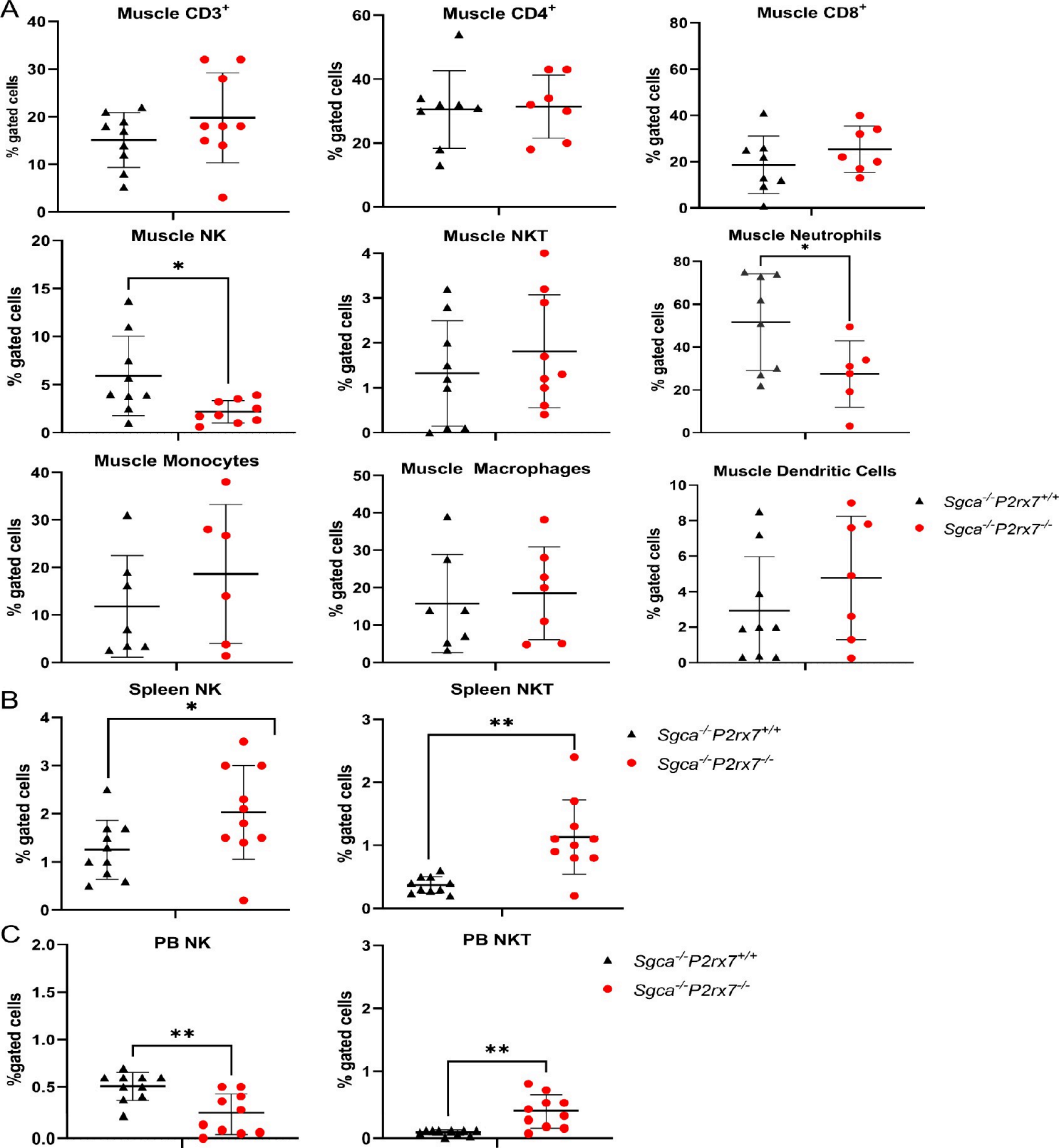
Evaluation of inflammatory cells in muscles, spleen, and peripheral blood. Flow cytometric analysis of immune cells isolated from (A) a pool of gastrocnemius, quadriceps, anterior tibialis, (B) spleen, and (C) peripheral blood excised from *Sgca* ^*–/–*^ *P2rx7* ^*+/+*^ (*n* = 10) and *Sgca* ^*–/–*^ *P2rx7* ^*–/–*^ (*n* = 10) mice. Immune cells were stained with specific antisurface markers: Ly6G, CD11b, F480, CD11c, CD3, CD4, CD8, CD25, and Foxp3. *p < 0.05; **p < 0.01.

Murine and human NK display a functional P2X7R. The importance of the receptor on NK activity has been studied mostly in tumor models.^35^ Still limited is the information on NK development, activation, trafficking, and homing in peripheral tissues. Blockade of P2X7R inhibits the sphingosine-1-phosphate signaling pathway, which is crucial for the egress in circulation of activated NK cells from bone marrow, spleen, and lymphonodes.^36,37^ Also, for NK, P2X7R could exert an indirect action through the involvement of other immune cells. It has been reported that the P2X7R expressed on dendritic cells mediates an IL-18-dependent NK activation in lung tumors.^38^ Notably, a significant reduction of NK population was observed in peripheral blood samples from *Sgca* ^*–/–*^ *P2rx7* ^*–/–*^ mice in comparison to *Sgca* ^*–/–*^ *P2rx7* ^*+/+*^ animals with a corresponding increase in the spleen (Figure 5B, C). This may indicate a defect in NK egression from the lymphoid organ, explaining a reduced number of NK cells infiltrating the muscle (Figure 5A), possibly including also the diaphragm. It may also be relevant to note that some NK subpopulations exert immunosuppressive effects by secreting IL-10.^39,40^ Thus, a lower percentage of immunosuppressive NK may concur with the aggravation of the inflammatory condition in *Sgca* ^*–/–*^ *P2rx7* ^*–/–*^ mice. A significant increase of NKT cells was detected both in the spleen and peripheral blood of *Sgca* ^*–/–*^ *P2rx7* ^*–/–*^ mice (Figure 5B, C), in line with the exquisite sensitivity of this cell subset to P2X7R-mediated cell death.^41^ None of the other immune cell populations was differentially abundant in the spleen or in peripheral blood (Supplementary Figure 6).

Immunofluorescence staining showed an increase in CD3^+^ lymphocytes and Iba1^+^ macrophages in *Sgca* ^*–/–*^ *P2rx7* ^*–/–*^ diaphragm sections compared to tissue from *Sgca* ^*–/–*^ *P2rx7* ^*+/+*^ mice (Figure 6A, B). In agreement with the histological analysis and acid phosphatase staining, no difference in the expression of inflammatory markers was observed in quadriceps from the two mouse strains (Figure 7A, B).

**Figure 6:**
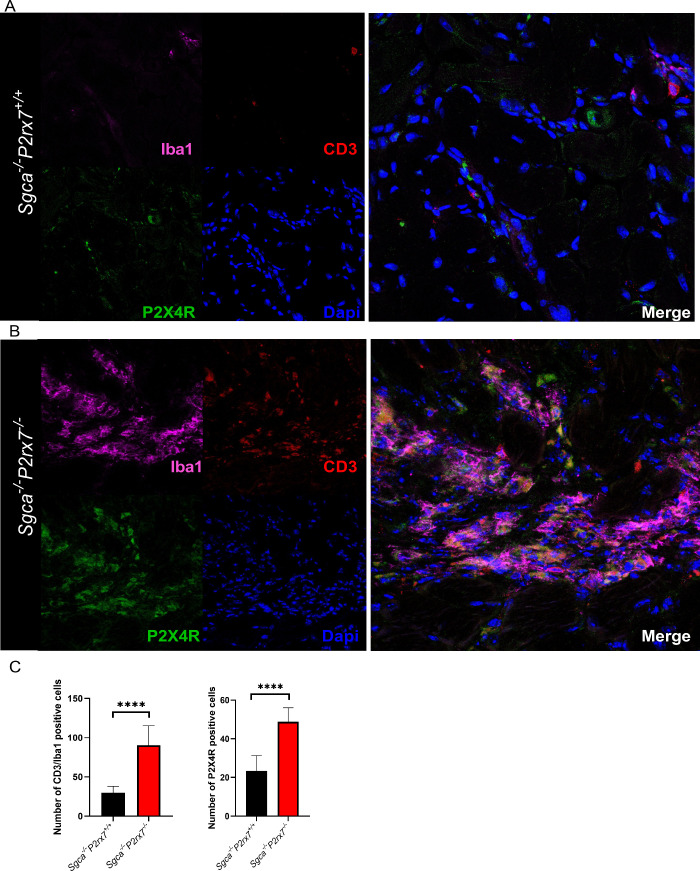
Evaluation of infiltrating immune cells and P2X4R expression. Representative images of immunofluorescence staining to reveal CD3 (marker for lymphocytes), Iba1 (marker for macrophages), and P2X4R expression in diaphragm sections from *Sgca* ^*–/–*^ *P2rx7* ^*+/+*^ (A) and *Sgca* ^*–/–*^ *P2rx7* ^*–/–*^ (B). Representative images are shown and quantification of inflammatory cells is reported in panel C. *n* = 2 images were acquired from 2 sections from *n* = 10 animals.

**Figure 7:**
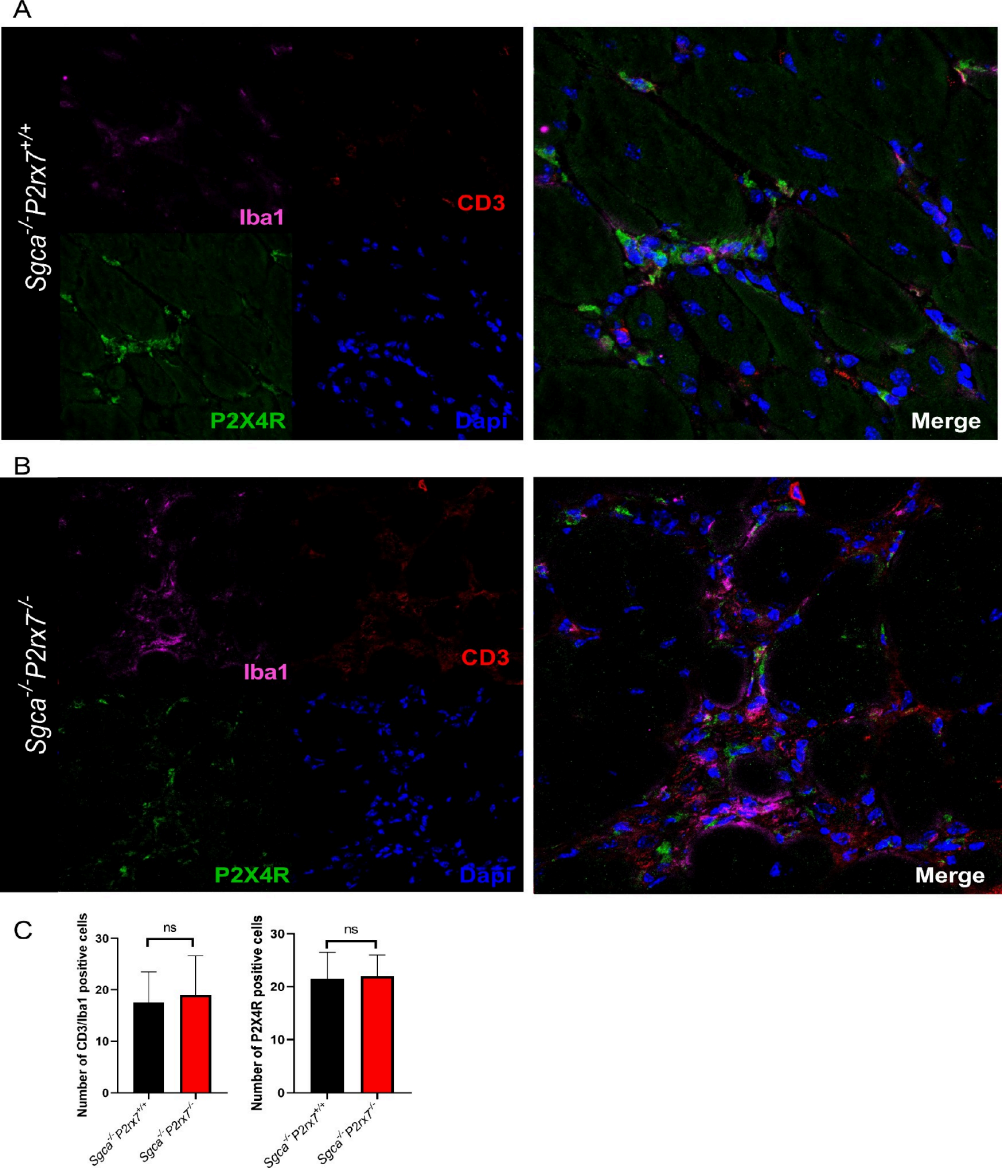
Evaluation of the infiltrating immune cells and P2X4R expression. Representative images of immunofluorescence staining to reveal CD3 (marker for lymphocytes), Iba1 (marker for macrophages), and P2X4R expression in quadriceps sections from *Sgca* ^*–/–*^ *P2rx7* ^*+/+*^ (A) and *Sgca* ^*–/–*^ *P2rx7* ^*–/–*^ (B). Representative images are shown and quantification of inflammatory cells is reported in panel C. *n* = 2 images were acquired from 2 sections from *n* = 10 animals.

Finally, the evaluation of muscle strength did not reveal differences between *Sgca* ^*–/–*^ *P2rx7* ^*–/–*^ and *Sgca* ^*–/–*^ *P2rx7* ^*+/+*^ mice. At 6, 12, 18, and 24 weeks of age, muscle strength was evaluated by the four-limb hanging test (Supplementary Figure 7). *Sgca* ^*–/–*^ *P2rx7* ^*–/–*^ mice exhibited progressively reduced functional performance, to a similar extent as *Sgca* ^*–/–*^ *P2rx7* ^*+/+*^, up to 18 or 24 weeks of age.

Our analysis confirmed a marked phenotypic difference between the diaphragm and limb muscles. This agrees with previous observations that found that in the *Sgca* ^*–/–*^ model, all the molecular and histological markers of muscular dystrophy are more pronounced in diaphragmatic tissue than in the limbs.^42^ Regenerative markers (myosin heavy chain 4, myogenin, myoblast determination protein 1), as well as molecules involved in fat deposition, such as peroxisome proliferator-activated receptor γ, Adipose Triglyceride Lipase 1, patatin like phospholipase domain containing 2 and hormone-sensitive lipase, fibrotic growth factors as transforming growth factor-Beta 1–3, and myostatin receptors myostatin/activin type I receptors (ALK4 and 5), are selectively increased in α-SG deficient diaphragms in comparison to quadriceps.^44^

### 2.3 Presence of P2X4R-Expressing Inflammatory Cells Confirms Increased Inflammation in Diaphragms from *Sgca* ^–/–^P2rx7^–/–^ Mice

The P2X4R is frequently coexpressed with P2X7R in different cell types, such as secretory epithelial and immune cells (macrophages, microglia, and monocytes). Since both receptors play a role in inflammatory processes,^43^ we also investigated P2X4R levels and localization in muscle tissue. The analysis in immunofluorescence revealed that P2X4R expression was increased in diaphragms (Figure 6A, B), but not in quadriceps (Figure 7A, B) of *Sgca* ^*–/–*^ *P2rx7* ^*–/–*^ mice, in comparison to *Sgca* ^*–/–*^ *P2rx7* ^*+/+*^ animals. Importantly, P2X4R colocalized with CD3 and Iba1 (Figures 6 and 7) and with CD45 (Supplementary Figure 8), suggesting that P2X4R was predominantly expressed by the infiltrating immune cells.

Spleen and thymus tissue isolated from WT mice were stained to highlight a positive CD3 staining (Supplementary Figure 9); in addition, the same tissues were isolated from both WT and *P2rx4* ^–/–^ mice and analyzed for positive P2X4R staining (Supplementary Figure 10).

P2X4R is a nonselective ATP cationic channel that can modulate inflammasome activation and trigger the secretion of inflammatory mediators, including the two pain mediators, prostaglandin E2 and brain-derived neurotropic factor.^44^ In T lymphocytes, P2X4R translocates rapidly to the immune synapse, formed by T cells and antigen-presenting cells, and facilitates productive T cell activation.^45^

In healthy muscle, P2X4R is expressed in vascular smooth muscle cells,^46^ in small- and medium-sized skeletal muscle vessels, whereas it is not detected in skeletal muscle myocytes.^14^ In muscular dystrophies, it is present in innate and adaptive immune cells.^16^

P2X7R and P2X4R receptors differ in their ATP sensitivity, with P2X4R having an intermediate sensitivity for ATP (EC50 ≅ 3–10 μM), while P2X7R is activated at higher concentrations of ATP (EC50 = 0.5–1 mM). P2X4R could contribute to P2X7R-mediated inflammation, although P2X4R triggers the inflammasome pathway also in the absence of P2X7R.^44,47^ In *Xenopus*, the two receptors can form heterotrimers,^43^ but recent data excluded a physical interaction on the plasma membrane of eukaryotic cells.^48^ Similarly, the two receptors can mutually regulate their expression, although different data have been collected in distinct cell and animal models.^48,49^

## 3 Conclusions

We have shown that contextual genetic *P2rx7* and *Sgca* deletion did not ameliorate the dystrophic phenotype in the proximal muscles of the lower limbs. It also did not affect the muscle myeloid subpopulations but induced a mild decrease in the number of infiltrating NK cells. The consequences of *P2rx7* deletion were more evident in diaphragms, the site of marked myofiber necrosis, where it enhanced the presence of inflammatory cells and strongly exacerbated the fibrotic replacement. These results are at odds with the amelioration of the dystrophic phenotype in α-sarcoglycan-null mice by broad-spectrum pharmacological P2X receptor antagonism.^16^ The present study further suggests that the outcome of genetic deletion of the *P2rx7* gene in knockout mice cannot be compared to the pharmacological P2X7R inhibition; in fact, P2X7R activity can condition immune effector responses as well as regenerative responses in a time and context-dependent fashion. Therefore, it should not be surprising that the combined deletion of *P2rx7* and *Sgca* genes resulted in a worsening dystrophic phenotype with respect to pharmacological P2X7R inhibition in dystrophic animals.

Inhibition of P2X7R activity or genetic deletion of *P2rx7* has been shown to reduce inflammatory markers and alleviate disease manifestations in various inflammatory and autoimmune conditions. Nonetheless, other studies have highlighted the possible detrimental effects of the lack of P2X7R in the complex pathogenesis of autoimmune disorders (reviewed in Grassi and Salina, ref 50). In the K/BxN murine model of autoimmune rheumatoid arthritis, P2X7R loss of function led to an increase in the number of T follicular helper (Tfh) cells in the intestinal Peyer’s patches (PPs), with resulting overproductive autoantibody responses and aggravation of the rheumatological phenotype.^51^ In fact, P2X7R limits Tfh cells' abundance in the PPs and was shown to restrict the expansion of pathogenic Tfh cells also in systemic lupus erythematosus.^52,53^ In the Myelin Oligodendrocyte Glycoprotein-induced model of autoimmune encephalopathy, *P2rx7* deletion led to a more severe pathology in the central nervous system than in *P2rx7* ^*+/+*^ animals, with more numerous and extended inflammatory regions and with enhancement of macrophage infiltration. A decreased rate of apoptosis of CD3^+^ cells surrounding the lesions was observed.^54^ In a model of chronic colitis, lack of P2X7R exacerbated intestinal fibrosis and increased collagen I expression, as well as M1 pro-inflammatory macrophage molecular markers.^55^ Finally, P2X7R-mediated cell death conditions the activity of unconventional T cells at the interface between innate and adaptive immunity.^28^ Altogether, these pieces of experimental evidence underline the importance of carefully considering the pleiomorphism and diffusion of P2X7R in a multitude of different cell types in the design of therapeutic strategies targeting the receptor. To date, clinical trials using P2X7R inhibitors have reached phase II for the treatment of rheumatoid arthritis and Crohn’s disease.^29,56,57^ These trials have indicated that further efforts will be needed to face challenges arising from the use of P2X7R inhibitors under immunopathological conditions. Finally, compensatory overexpression of P2X4R, or other purinergic receptor(s), in immune cells, as well as a direct regulatory action of P2X7R on fibrotic genes in fibroadipogenic precursors in strongly inflamed and profibrotic microenvironments, should be considered. Indeed, P2X7R has been demonstrated to regulate collagen expression in intestinal fibroblasts.^55^ Also, although our findings indicate that P2X7R is not expressed on Pax7^+^ quiescent cells, P2X7R might be turned on upon muscle damage and have a role in muscle stem cell activation and differentiation in myoblasts. Cell-type and time-specific conditional knockouts of the gene can unveil the intrinsic or extrinsic factors determining the phenotype. Overall, our results support a cautious assessment of timing, duration, and dosage of the treatment in the translation to the clinic of P2X7R antagonists.

## 4 Methods

### 4.1 In Vivo Experiments

To generate the *Sgca* ^*–/–*^ *P2rx7* ^*–/–*^ double knock out mouse model *Sgca* ^*–/–*^ mice were crossed with B6.129P2-*P2rx7* ^tm1Gab^/J (JAX #:005576) mice.

*Sgca* ^*–/–*^ *P2rx7* ^*+/+*^ (*n* = 10) and *Sgca* ^*–/–*^ *P2rx7* ^*–/–*^ (*n* = 10) were bred in the Animal Facility at Policlinico San Martino, Genova. All mice were housed under standard specific pathogen-free conditions and allowed access to food and water ad libitum. Animals were euthanized at 24 weeks of age by carbon dioxide inhalation. All experimental protocols were approved by the Policlinico San Martino Animal Welfare Body and by the Italian Ministry of Health (Authorization number 1063-2020-PR). The health status of the animals was monitored daily.

Twenty-week-old Tg:Pax7-*n*GFP (*n* = 3) were kindly donated by Prof. Dentice (Department of Clinical Medicine and Surgery, University of Naples “Federico II”, Naples, Italy).

### 4.2 Histological Analysis

Diaphragms and quadriceps isolated from *Sgca* ^*–/–*^ *P2rx7* ^*+/+*^ and *Sgca* ^*–/–*^ *P2rx7* ^*–/–*^ mice and biopsies taken from quadriceps of control subjects or of patients affected by LGMDR3 were cut on cryostat (Leica Biosystems, Deer Park, IL, USA), and 7-μm-thick sections were stained with standard hematoxylin and eosin (H&E) (reagents from Sigma-Aldrich), Masson trichrome staining (reagents from Carl Roth, Karlsruhe, Germany and from Merck) to evaluate muscle fibrosis and acid phosphatase (reagents from Merck) to detect inflammatory reactions. Representative pictures were taken using a Nikon Ti Eclipse microscope at 10× or 20× magnification. Quantification of trichrome staining was performed using semiautomated measurement tools in NIS-Elements AR software version 4.20 and expressed in terms of percentage stained area of the total section area. Whole slide images were acquired using the Manual WSI software version 2020C-34FL (Microvisioneer, Esslingen am Neckar, Germany). The histological sections of *Sgca* ^*–/–*^ *P2rx7* ^*+/+*^ and *Sgca* ^*–/–*^ *P2rx7* ^*–/–*^ mice displaying freezing artifacts were not analyzed.

### 4.3 Immunofluorescence Staining

Unfixed sections (7-μm-thick) of quadriceps and diaphragms of *Sgca* ^*–/–*^ *P2rx7* ^*+/+*^ and *Sgca* ^*–/–*^ *P2rx7* ^*–/–*^ mice, and of tibialis anterior of *Tg:Pax7-nGFP* mice and human samples of control subjects and patients affected by LGMDR3 were incubated with a blocking solution containing 0.2% TritonX-100 (Sigma-Aldrich), 2% bovine serum albumin (Sigma-Aldrich), and 5% fetal bovine serum (GIBCO, Thermo Fisher Scientific), 2% goat serum (GIBCO) in PBS for 1 h at room temperature (RT). Sections were afterward incubated for 18 h at 4 °C in a humified chamber with the following primary antibodies: anti-CD3 (dilution 1:500, Abcam), anti-Iba1 (dilution 1:500, Synaptic Systems GmbH, Göttingen, Germany), anti-CD45 (dilution 1:25), anti-P2X7R (dilution 1:200 Alomone Laboratories, Jerusalem, Israel for human samples; dilution 1:400, Synaptic Systems GmbH for murine samples), anti-P2X4R (dilution 1:400, Alomone Laboratories) and anti-GFP (dilution 1:500, Abcam). Slides were washed 3 times for 5 min with PBS and incubated for 1 h at room temperature with the appropriate fluorescent dye-conjugated secondary antibodies diluted in 0.2% BSA (Sigma-Aldrich) and, only for slices of tibialis anterior of *Tg:Pax7-nGFP* mice, with YO-PRO-1 (dilution 1:1000). After washing (3 times for 5 min with PBS), slices were incubated for 3 min with 4′,6-diamidino-2-phenylindole (DAPI, 0.1 mg/L in PBS) and washed again (2 times for 10 min with PBS). Slides were mounted using PermaFluor mounting medium (Epredia, Kalamazoo, MI, USA) and were kept overnight at RT and then stored at 4 °C until confocal acquisition using a Zeiss LSM 880 (Carl Zeiss, Oberkochen, Germany) or Nikon AX R (Tokyo, Japan). Samples were analyzed with the same microscope settings. For long-term storage, slices were kept at −20 °C.

### 4.4 Flow Cytometry

Hematopoietic cells were collected from peripheral blood (PB), limb muscles, and spleen. Cells collected from PB were first incubated with 2 μL/sample of TruStainFcXTM antimouse CD16/32 for 5 min in order to block Fc receptor and then with cocktails of antibodies specific for CD45, CD3, CD4, CD8, CD11b, CD11c, CD25, F4/80, Ly-6C, and Ly-6G for 30 min. After antibody incubation, samples were lysed (Becton Dickinson Pharm Lyse TM, San Josè, CA, USA), washed, and resuspended in 300 μL of PBS. All the antibodies were purchased from Biolegend (San Diego, CA, USA). Gastrocnemius, quadriceps, and anterior tibialis excised from *Sgca* ^*–/–*^ *P2rx7* ^*+/+*^ and *Sgca* ^*–/–*^ *P2rx7* ^*–/–*^ mice were resuspended in RPMI 1640 base medium (Euro Clone, Milan, Italy), mechanically and enzymatically digested using skeletal muscle dissociation kit (Miltenyi Biotec, Bologna, Italy) and filtered through 100- and 70-μm mesh filters (BD Bioscience, San Jose, CA, USA). After filtration, cells were purified using gradient centrifugation by Percoll solution (GE Healthcare Biosciences, Uppsala, Sweden) and stained with Live/DeadTM Fixable Yellow Dead Cell Stain Kit (Invitrogen, Thermo Fisher Scientific) and the antibodies listed above. The spleen was mechanically digested, filtered through 100 and 70 μm mesh filters, counted, and stained as described for PB and muscle.

All acquisitions were performed with a three laser LSR Fortessa X20 (Becton Dickinson), and the obtained FSC files were analyzed with Kaluza Software (version 2.1, Beckman Coulter). The immune profile of the peripheral blood, spleen, and muscles was performed in the same animals.

### 4.5 Myoblasts Isolation and FACS Analysis

Myoblasts were isolated from 3-week-old WT and *Sgca* ^*–/–*^ *P2rx7* ^*+/+*^ puppies. Forelimb, hind limb, and diaphragm muscles were dissected, mechanically cut, and enzymatically digested at 37 °C under constant shaking with a solution containing Liberase DL (2.5 mg/mL; Roche, Basel, Switzerland) and DNase I (100 μg/mL; Roche, Basel, Switzerland) in PBS (Merck). Undigested tissue was precipitated for 5 min; supernatants were passed through 100 and 40 μm filters and then centrifuged for 5 min at 250 × *g*. Cell pellets were resuspended in Dulbecco’s modified Eagle’s medium (DMEM) plus 20% fetal bovine serum, 10% horse serum, 1% l-glutamine, 1% penicillin/streptomycin, and basic fibroblast growth factor 2.5 ng/mL (Gibco); next, cells were preplated in 100 mm uncoated Petri dishes for 1 h. After preplating, the nonadherent satellite cell-enriched population was collected and plated in gelatin-coated (Gelatin Type A: from Porcine Skin; Merck) 100 mm Petri dishes at a density of 30,000 cells per Petri dish. After 5 days in proliferation, the isolated myoblasts were analyzed by flow cytometry. Cells were stained with Live/Dead Fixable Yellow Dead Cell Stain Kit (Invitrogen, Thermo Fisher Scientific) and CD56 BV421 (BD Biosciences), and antimurine P2X7R (Alomone), followed by the secondary goat antirabbit APC conjugated antibody (Abcam). As a positive control for anti-P2X7R staining, the murine BV2 cell line was used: microglia cells were cultured in RPMI 1640 (Euroclone, Milan, Italy) medium, containing 10% Fetal Bovine Serum (FBS; Euroclone) and 1% penicillin-streptomycin solution (100×; Euroclone), at 37 °C and 5% CO_2_.

### 4.6 Four-Limb Hanging Test

At 4, 12, and 24 weeks of age, the muscle strength of *Sgca* ^*–/–*^ *P2rx7* ^*+/+*^ and *Sgca* ^*–/–*^ *P2rx7* ^*–/–*^ mice was scored through the four-limb hanging test. Mice were subjected to a 180 s lasting hanging test, during which a falling score was recorded. The animals had to hang for three trials, and the average maximum hanging time of the three trials was measured (standard operating procedure).^58^

### 4.7 Statistical Analysis

Statistical parameters, including the exact value of *n* and statistical significance, are reported in the figures and their associated legends. Data distribution was verified using the normality test, and results were analyzed using the unpaired *t*-test, using GraphPad Prism 3.0 software (GraphPad Software, El Camino Real, San Diego, CA, USA).

### 4.8 Ethics

All human samples were collected after patients had signed informed consent forms in accordance with the requirements of the G. Gaslini Institute Ethics Committee.

## Supplementary Material



## Data Availability

Data will be made available on request.

## Notes

Supplementary Table 1: patients; Supplementary Figure 1: evaluation of P2X7R expression in biopsies of dystrophic muscles of *Sgca* ^*–/–*^ mice: nuclei and IgG control; Supplementary Figure 2: evaluation and quantification of P2X7R and CD45 expression in biopsies of dystrophic muscles of *Sgca* ^*–/–*^ mice; Supplementary Figure 3: evaluation by flow cytometry of P2X7R expression on cultured myoblasts; Supplementary Figure 4: evaluation of P2X7R expression in satellite cells; Supplementary Figure 5: evaluation of P2X7R expression in biopsies of skeletal muscle from patients affected by LGMDR3; Supplementary Figure 6: evaluation of inflammatory cells in spleen and peripheral blood; Supplementary Figure 7: evaluation of motor performance; Supplementary Figure 8: evaluation and quantification of P2X4R and CD45 expression in biopsies of dystrophic muscles of *Sgca* ^*–/–*^ mice; Supplementary Figure 9: evaluation of CD3 expression in biopsies of the spleen and thymus of wild type mice; and Supplementary Figure 10: evaluation of the P2X4R expression in biopsies of the spleen and thymus of wild-type and *P2rx4* ^–/–^ mice (PDF)
