# Supplementary material for: Genetic Deletion of the Purinergic Receptor P2rx7 Worsens the Phenotype of α‑Sarcoglycan Muscular Dystrophy
Source: ACS Pharmacol Transl Sci. 2025 Sep 11;8(10):3477–89. doi: 10.1021/acsptsci.5c00138 (PMC12519300; doi:10.1021/acsptsci.5c00138)
Supplement: Supplementary file 1 [file pt5c00138_si_001.pdf]

Supporting Information

Genetic deletion of the purinergic receptor *P2rx7* worsens the phenotype of a-Sarcoglycan Muscular Dystrophy

Cecilia Astigiano[1,§], Elisa Principi[2,§], Sara Pintus[2,3,§], Andrea Benzi[1], Serena Baratto[2], Chiara Panicucci[2], Mario Passalacqua[1], Juan Sierra-Marquez[4], Annette Nicke[4], Francesca Antonini[5], Genny Del Zotto[5], Annunziata Gaetana Cicatiello[6], Lizzia Raffaghello[7], Tanja Rezzonico Jost[8], Fabio Grassi[9,10], Santina Bruzzone[1,11], Claudio Bruno[2,12,†,*], Elisabetta Gazzerro[13,†,*]

[1]Department of Experimental Medicine, Section of Biochemistry, University of Genoa, 16132 Genoa, Italy;
[2]Center of Translational and Experimental Myology, IRCCS Istituto Giannina Gaslini, 16147 Genoa, Italy;
[3]Laboratory of Gene Expression Regulation, IRCCS Ospedale Policlinico San Martino, 16132 Genova, Italy;
[4]Walther Straub Institute of Pharmacology and Toxicology, Faculty of Medicine, LMU Munich, 80336 Munich, Germany;
[5]Core facilities Department of Research and Diagnostics, IRCCS Istituto G. Gaslini, 16147 Genoa, Italy;
[6]Department of Clinical Medicine and Surgery, University of Naples "Federico II", Naples, Italy;
[7]Molecular Oncology and Angiogenesis Unit, IRCCS Ospedale Policlinico San Martino, 16132 Genova, Italy;
[8]Institute of Oncology Research (IOR), 6500 Bellinzona, Switzerland;
[9]Istituto Nazionale Genetica Molecolare "Romeo ed Enrica Invernizzi", Milan, Italy;
[10]Department of Medical Biotechnology and Translational Medicine, University of Milan, 20133 Milan, Italy;
[11]IRCCS Ospedale Policlinico San Martino, 16132 Genova, Italy;
[12]Department of Neurosciences, Rehabilitation, Ophthalmology, Genetics, Maternal and Child Health (DINOGMI), University of Genova, 16132 Genova, Italy
[13]Unit of Muscle Research, Experimental and Clinical Research Center, Charité Universitätsmedizin and Max Delbrück Research Center for Molecular Medicine, 10627 Berlin, Germany.

§co-first authors

†co-last and co-corresponding

*corresponding Authors: Elisabetta Gazzerro, elisabetta.gazzerro@charite.de; Claudio Bruno, claudiobruno@gaslini.org

Supplementary Table 1

Patient	Sex	Mutation Allele 1	Mutation Allele 2	Onset (years)	MB (years)	α-SG membrane expression	Inflammatory infiltrates
1	F	c.739G>A; p.Val247Met	c.850C>T; p.Arg284Cys	10.0	10.0	slightly reduced	low
2	M	c.409 G>A; p.Glu137Lys	c.739 G>A; p.Val247Met	8.6	26.0	slightly reduced	low
3	F	c.229C>T; p.Arg77Cys	IVS5: c.584+5G>A	0.7	0.8	absent	high
4	M	c.89_delC P30HfsX16	c.89_delC P30HfsX16	4.0	2.0	markedly reduced	high

Abbreviations. MB: Muscle biopsy.

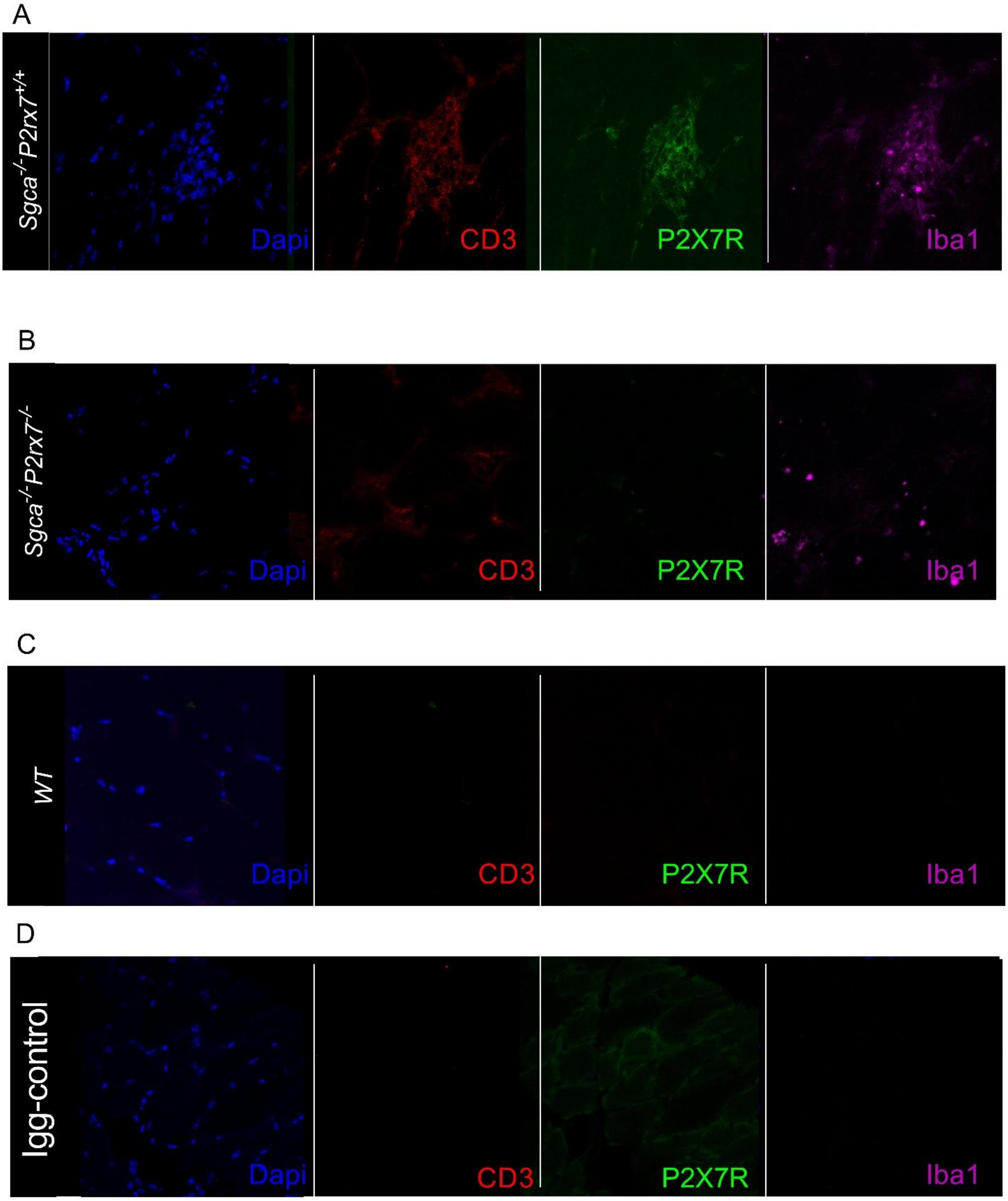

Supplementary Figure 1

Evaluation of P2X7R expression in biopsies of dystrophic muscles of *Sgca*$^{-/-}$ mice: nuclei and IgG control. Representative images of immunofluorescence staining to localize P2X7R, CD3 and Iba1 in skeletal muscle (quadriceps) from *Sgca*$^{-/-}$*P2rx7*$^{+/+}$ (A) *Sgca*$^{-/-}$*P2rx7*$^{-/-}$ (B) and WT (C) mice. D, IgG control. *n*=3 images were acquired from 2 slices obtained from *n*=3 animals. 40x Magnification

A Supplementary Figure 2A *Sgca*$^{-/-}$*P2rx7*$^{+/+}$ mice

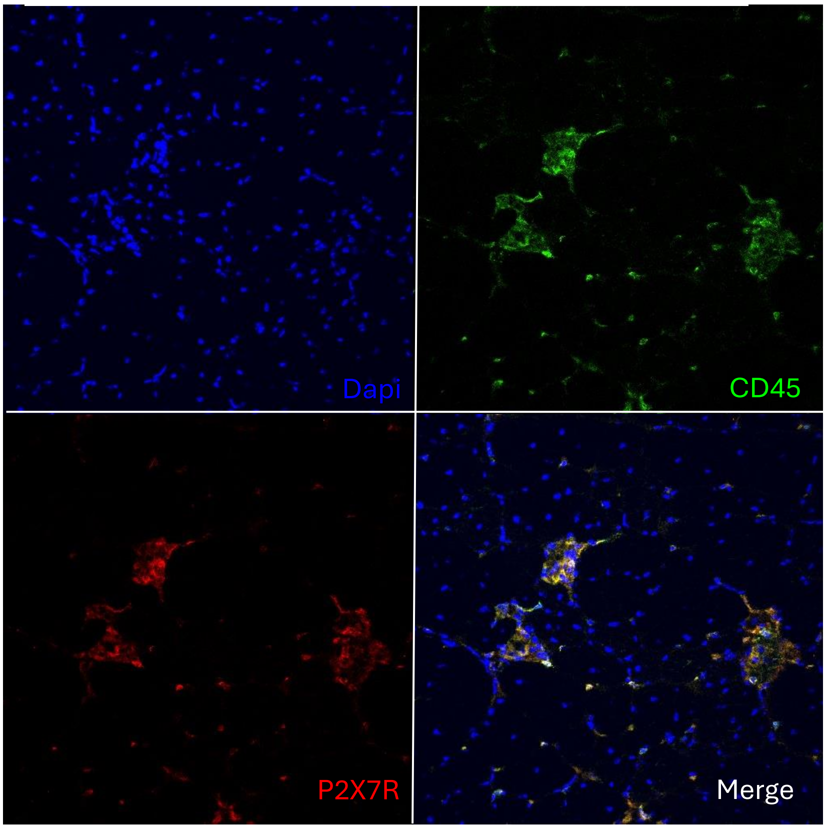

#1 mouse

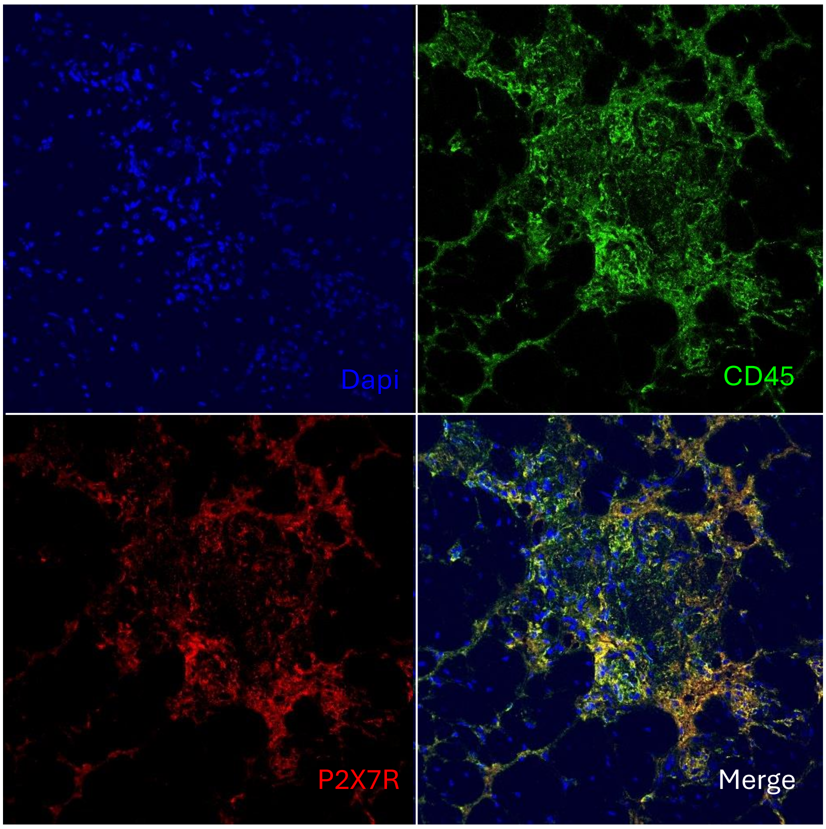

#2 mouse

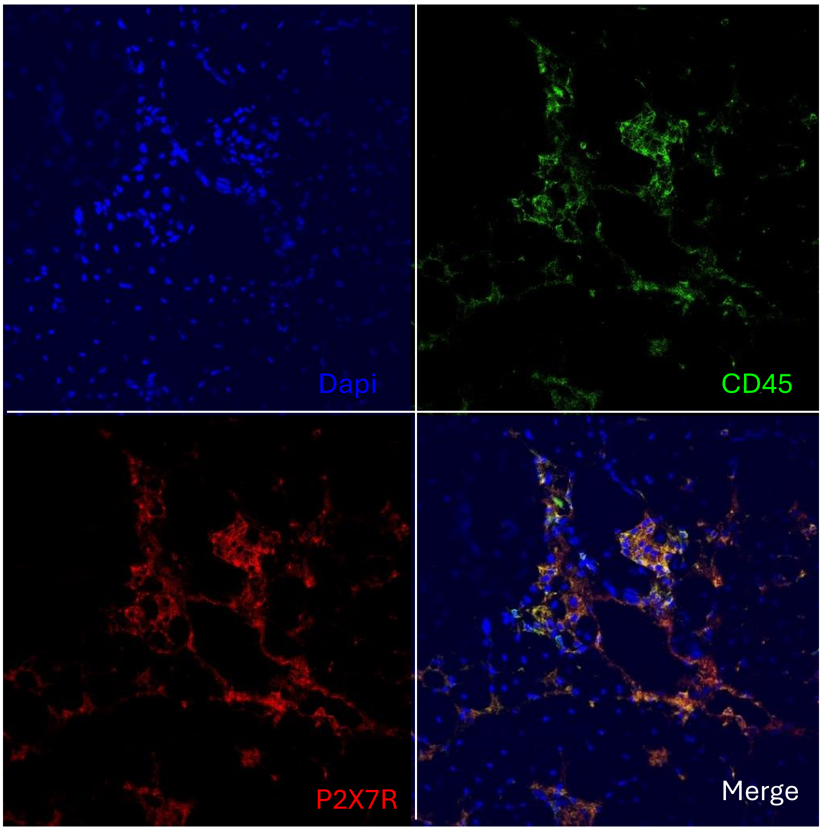

#3 mouse

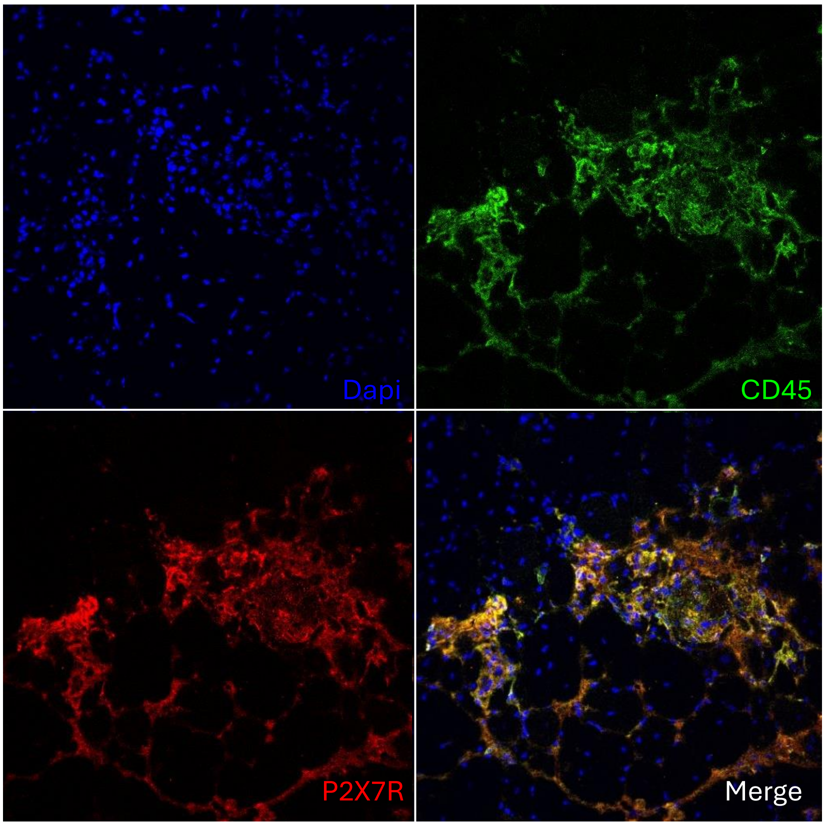

#4 mouse

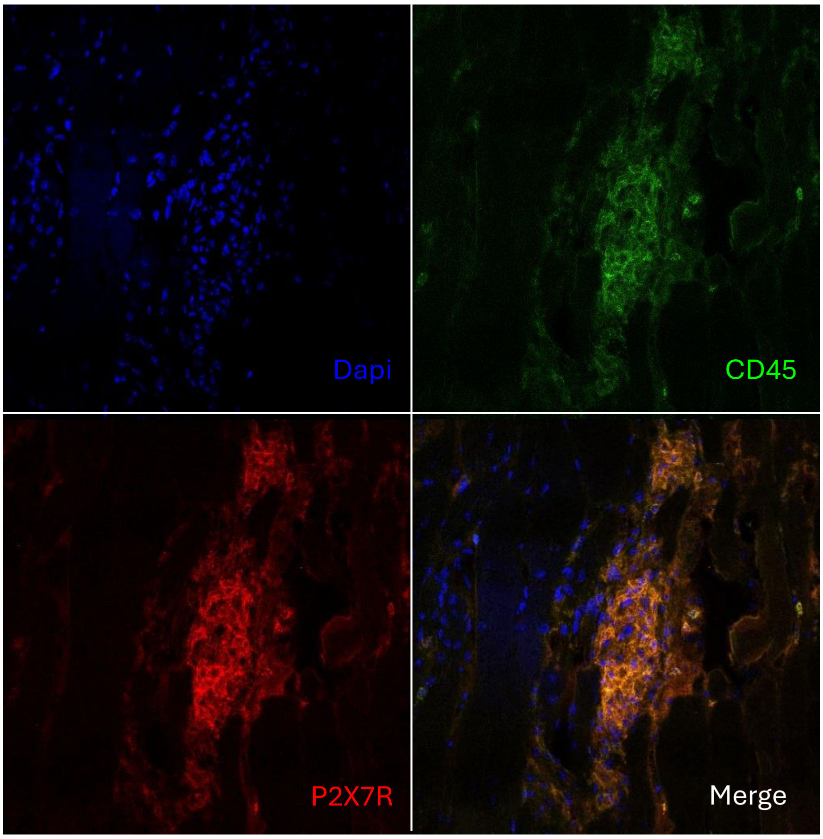

#5 mouse

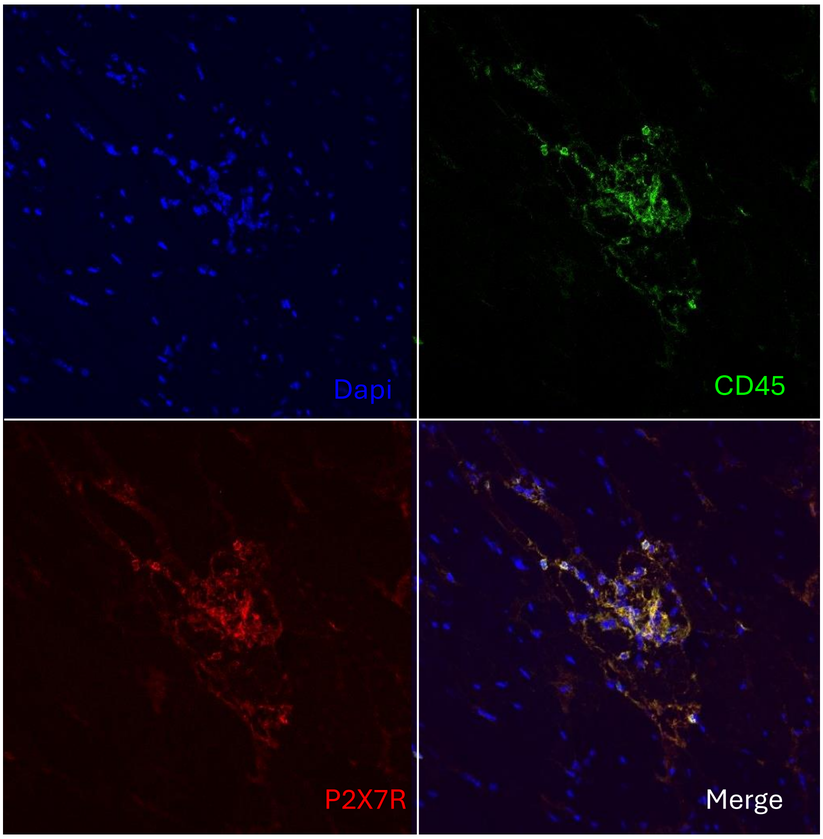

#6 mouse

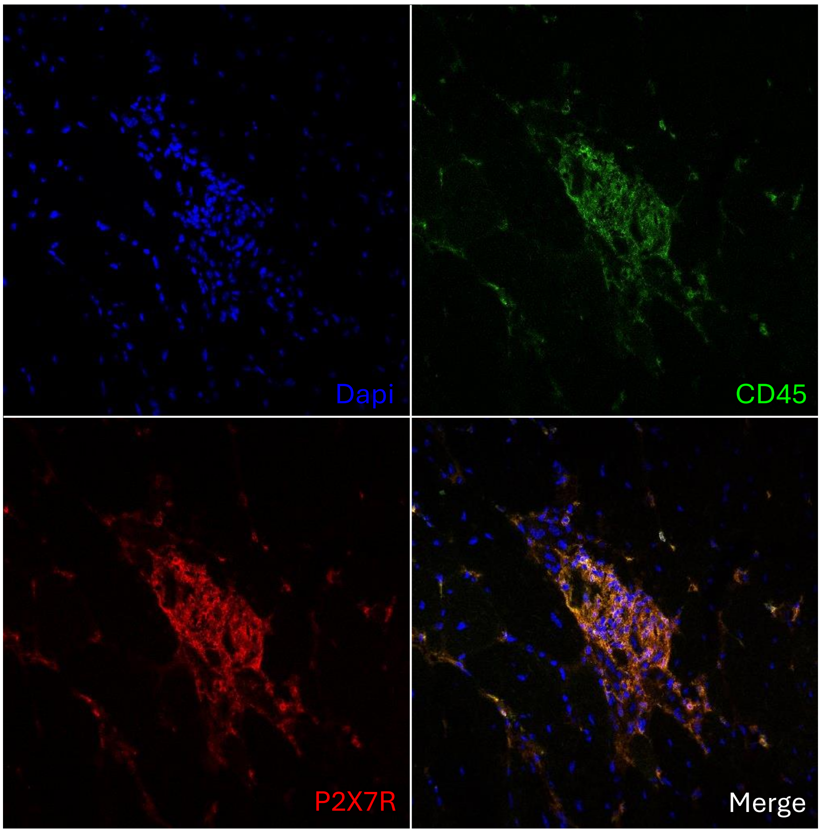

#7 mouse

B Supplementary Figure 2B *Sgca*$^{-/-}$*P2rx7*$^{-/-}$

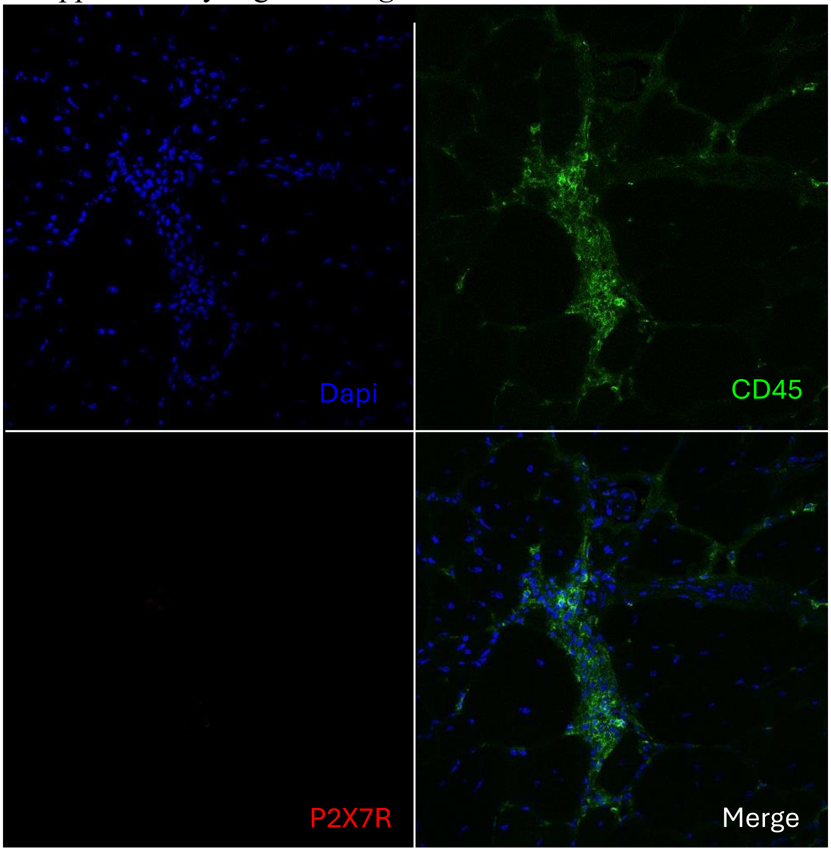

#1 mouse

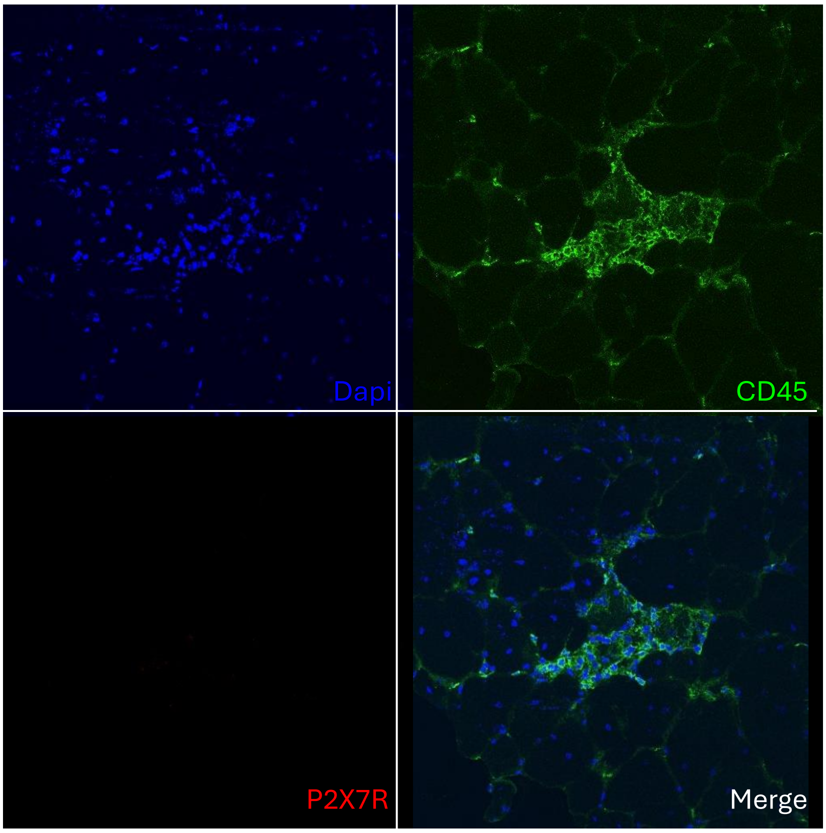

#2 mouse

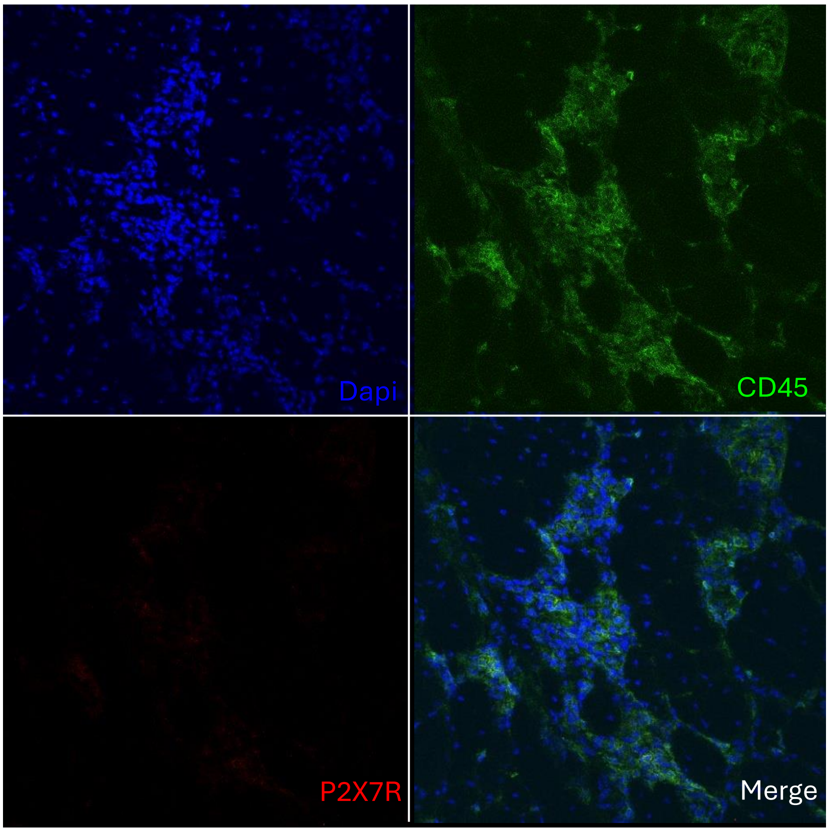

#3 mouse

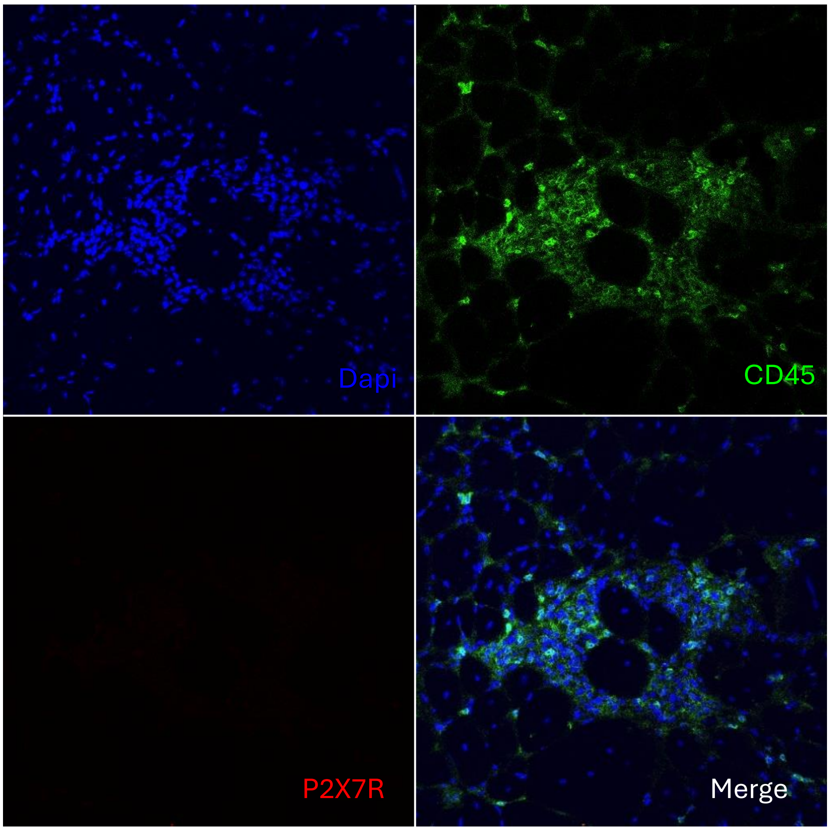

#4 mouse

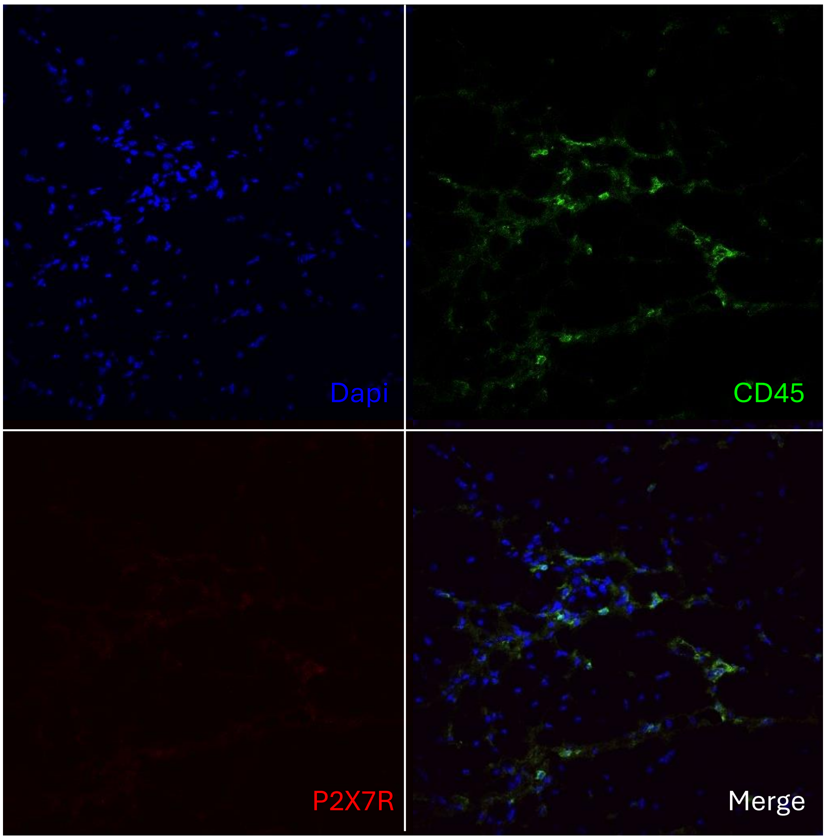

#5 mouse

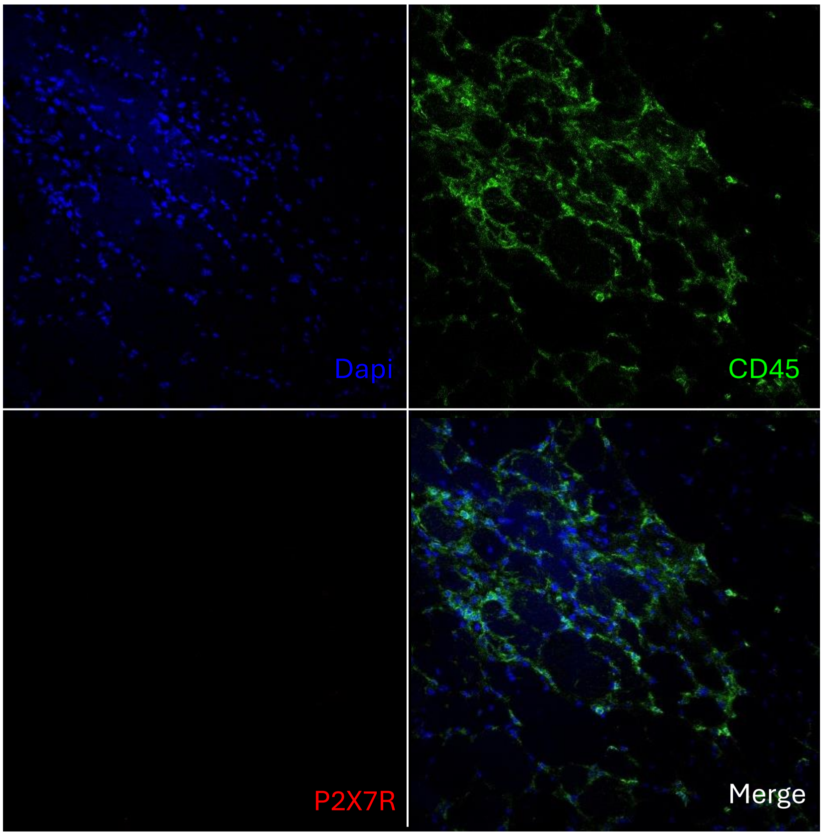

#6 mouse

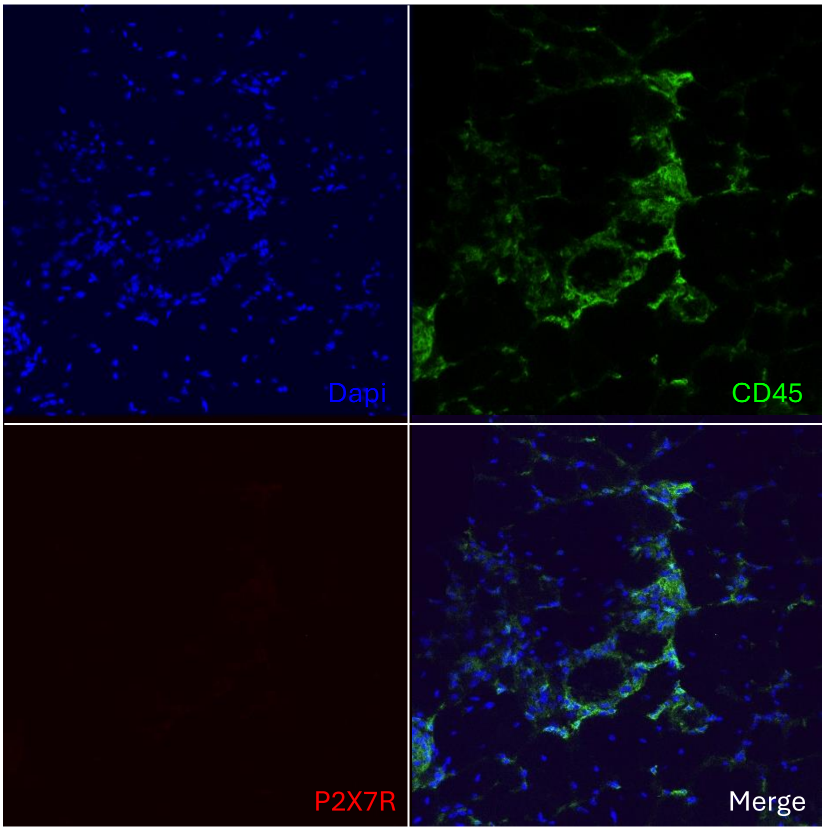

#7 mouse

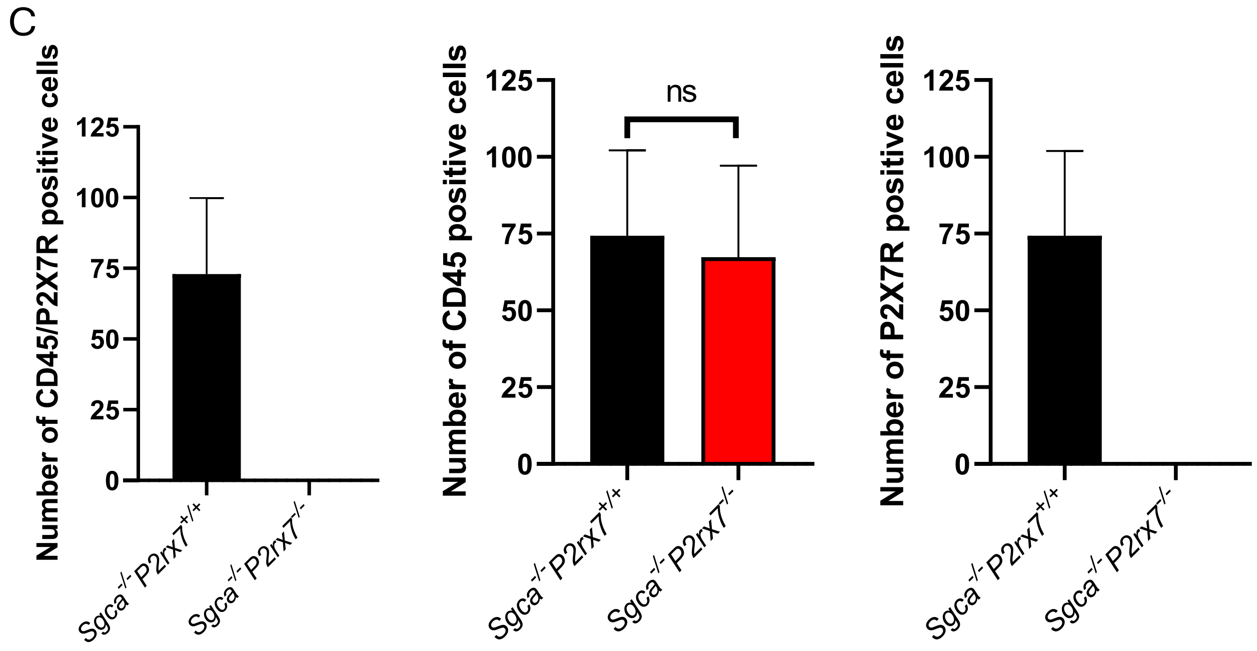

Supplementary Figure 2

Evaluation and quantification of P2X7R and CD45 expression in biopsies of dystrophic muscles of *Sgca*$^{-/-}$ mice. Representative images of immunofluorescence staining to localize P2X7R and CD45 in skeletal muscle (quadriceps) from *Sgca*$^{-/-}$*P2rx7*$^{+/+}$ (A) *Sgca*$^{-/-}$*P2rx7*$^{-/-}$ (B); 40X Magnification. (C) Quantification of the number of CD45^{+} and P2X7R^{+} cells (left panel), only CD45^{+} cells (middle panel), only P2X7R^{+} cells (right panel). *n*=2 images were acquired from 2 slices obtained from *n*=7 animals per each genotype. ns, not statistically different.

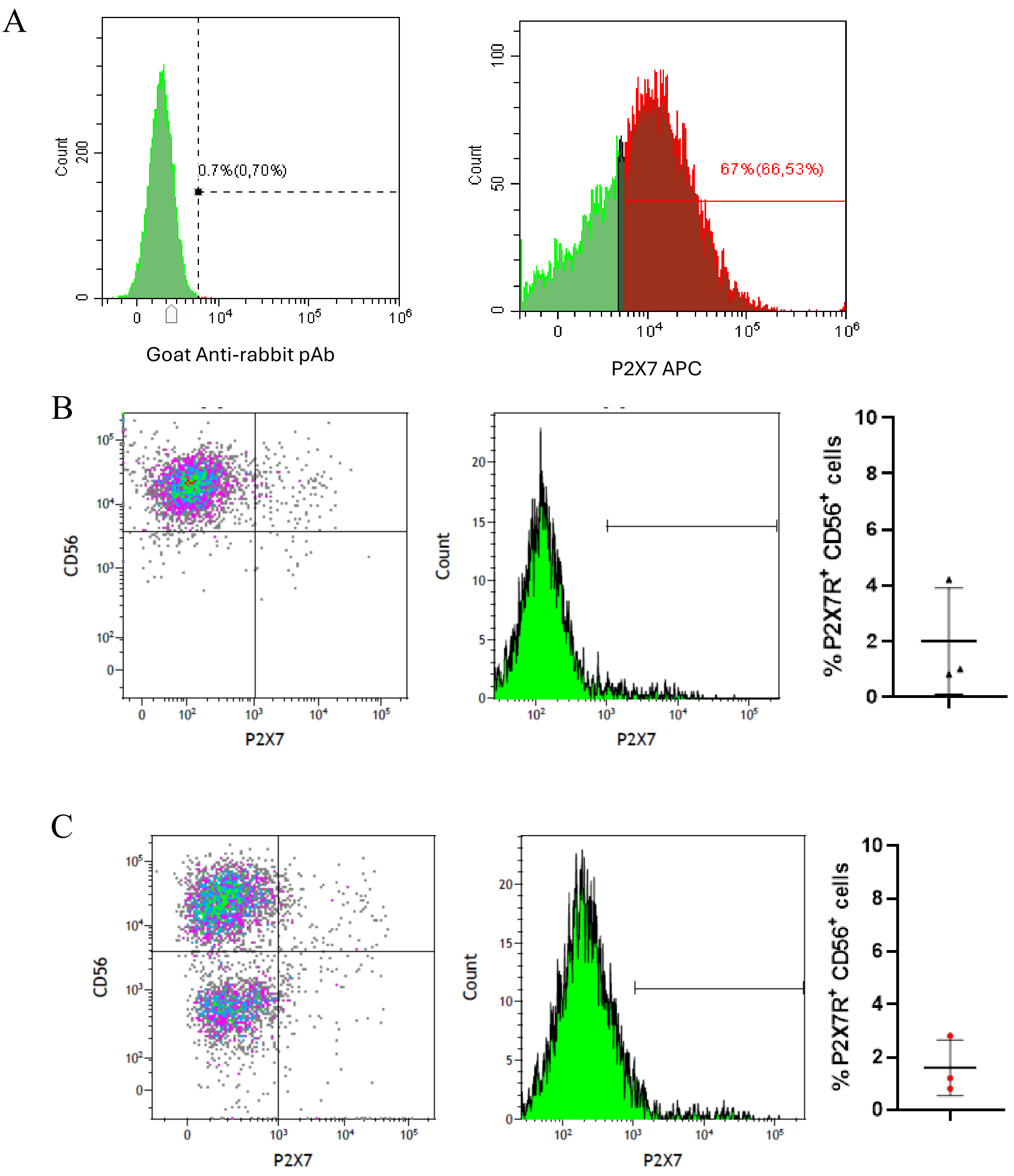

Supplementary Figure 3

Evaluation by flow cytometry of P2X7R expression on cultured myoblasts. Flow cytometric analysis to evaluate P2X7R and CD56 expression on: (B) myoblasts isolated from skeletal muscles of WT mice (n = 3) and (C) myoblasts isolated from skeletal muscles of *Sgca*$^{-/-}$ (n = 3) animals. (A) Positive control for the anti-P2X7R antibody in P2X7R^{+} murine microglia cell line (BV2 cells).

A

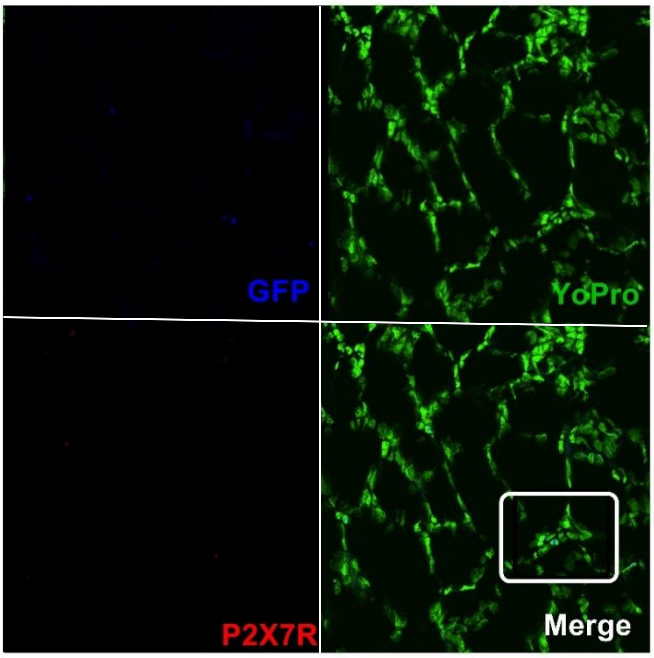

GFP
YoPro
P2X7R
Merge

B

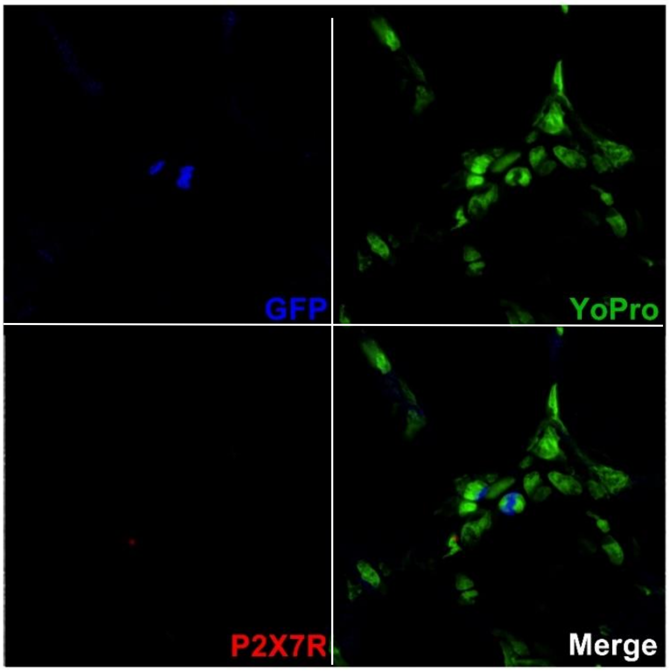

GFP
YoPro
P2X7R
Merge

C

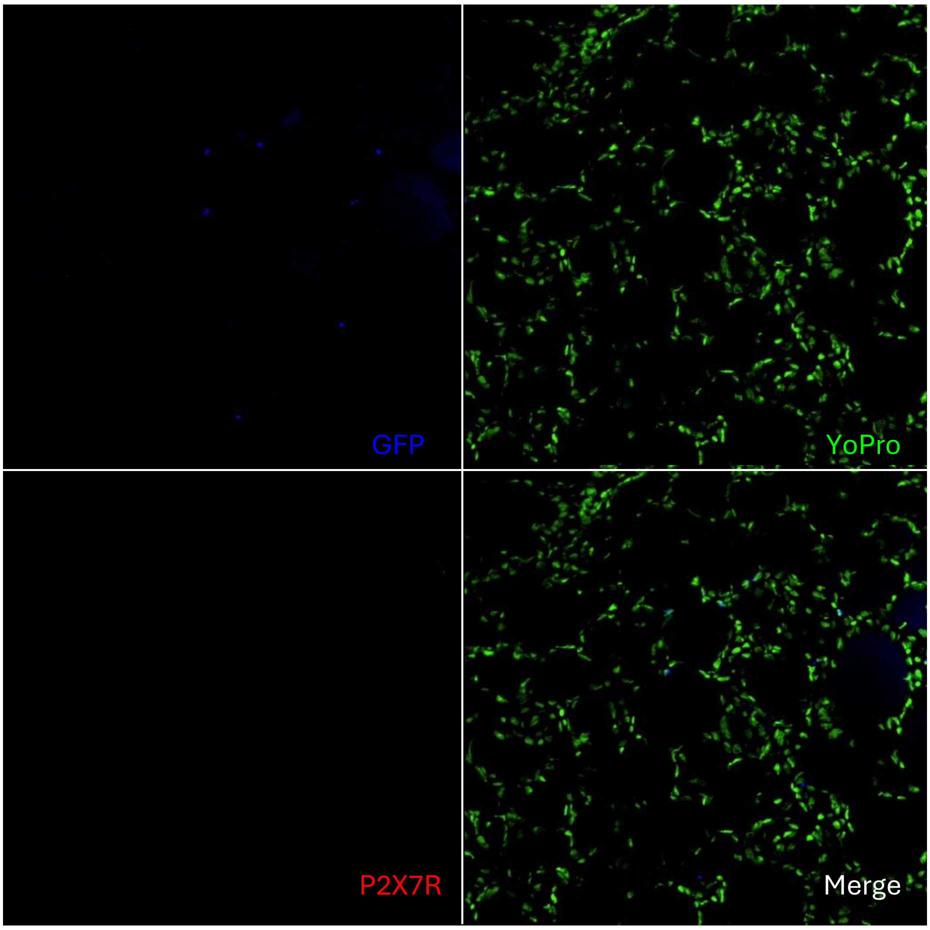

GFP
YoPro
P2X7R
Merge

#1 image

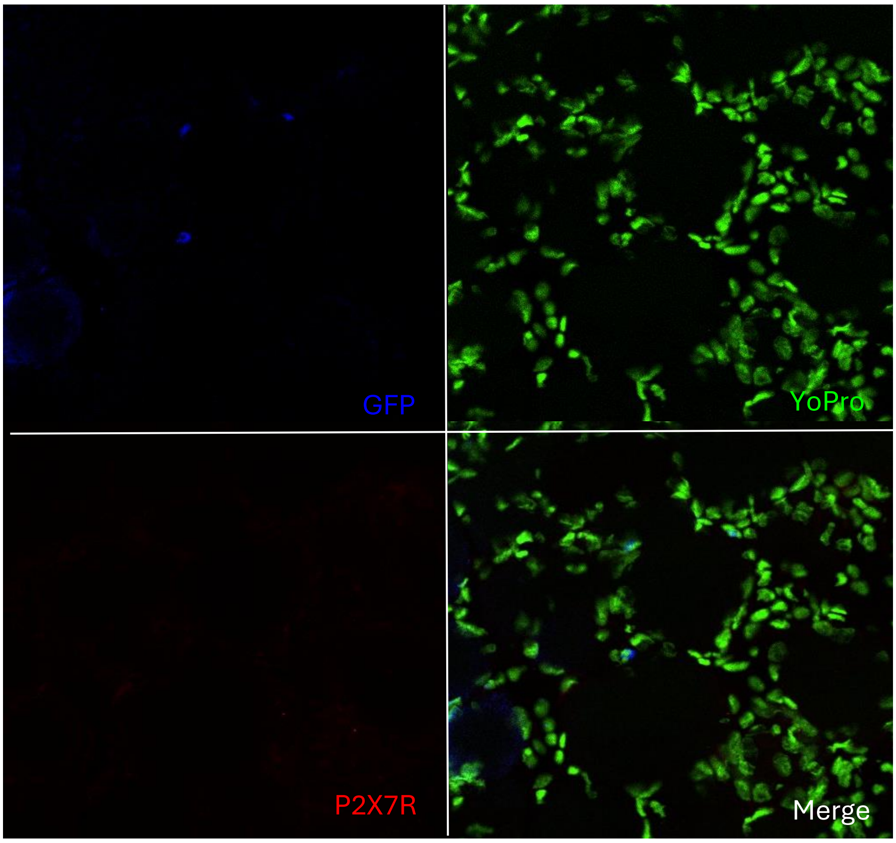

#2 image

Supplementary Figure 4

Evaluation of P2X7R expression in satellite cells. Representative images of immunofluorescence staining to reveal GFP (Pax7$^+$ cells) and P2X7R expression in tibialis anterior muscle sections from Tg:Pax7-nGFP mice. Panel B shows a 60X magnification of the indicated area in panel A (40X). (C) Additional representative images at different 20X (image 1) and 40X (image 2) magnification of Pax7$^+$ cells. *n*=3 images were acquired from 2 slices.

A

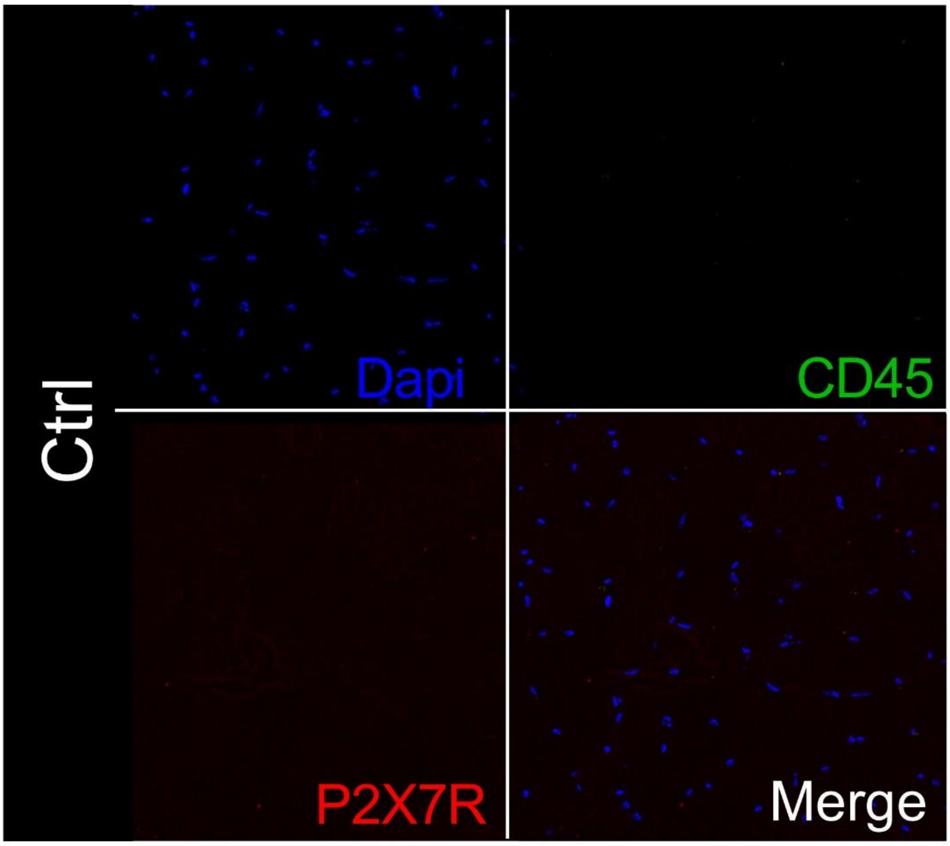
Ctrl
Dapi
CD45
P2X7R
Merge

B

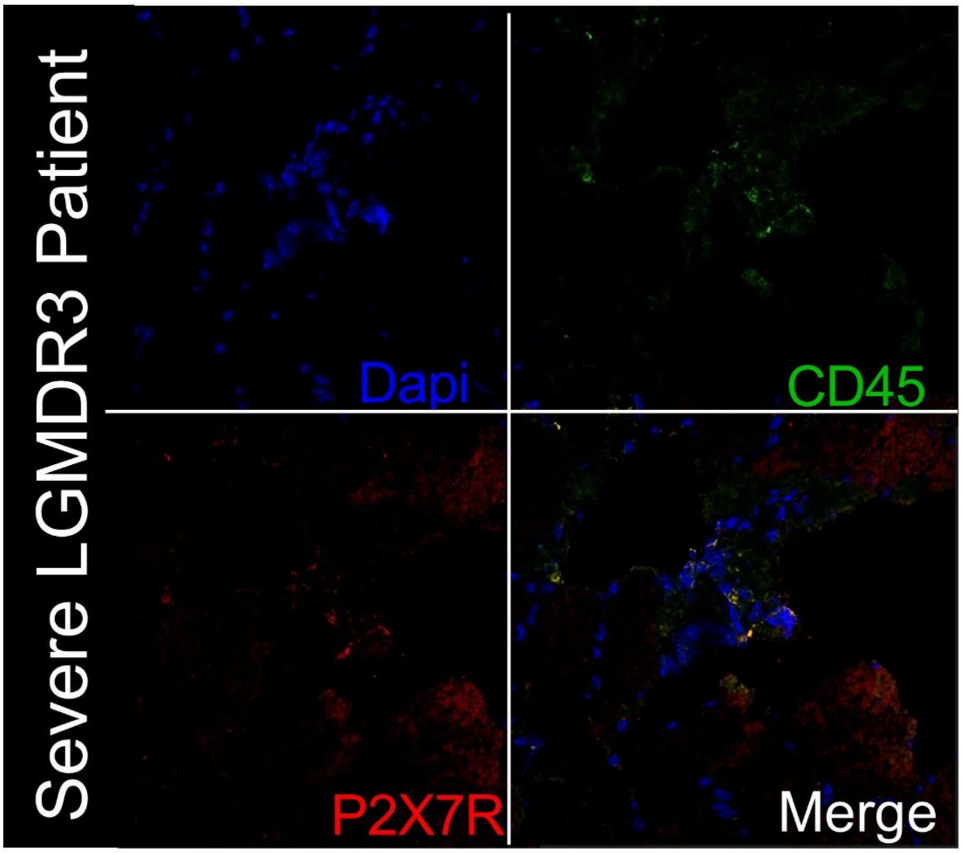
Severe LGMDR3 Patient
Dapi
CD45
P2X7R
Merge

#1 patient

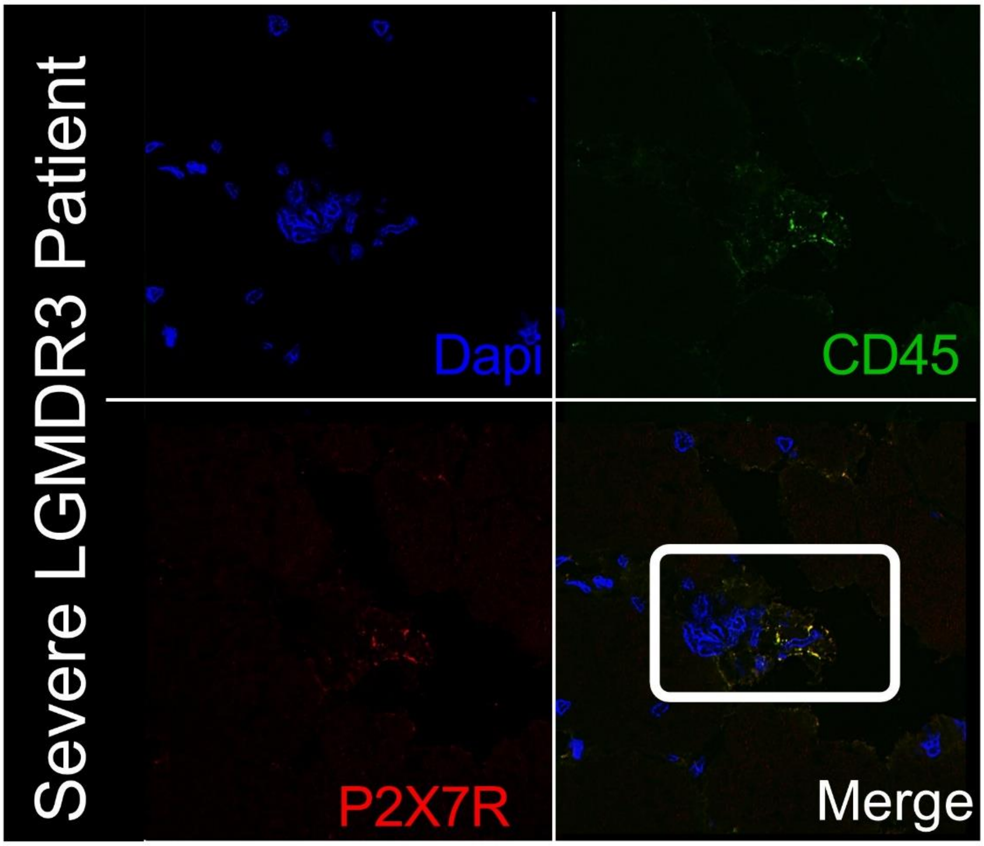

#2 patient

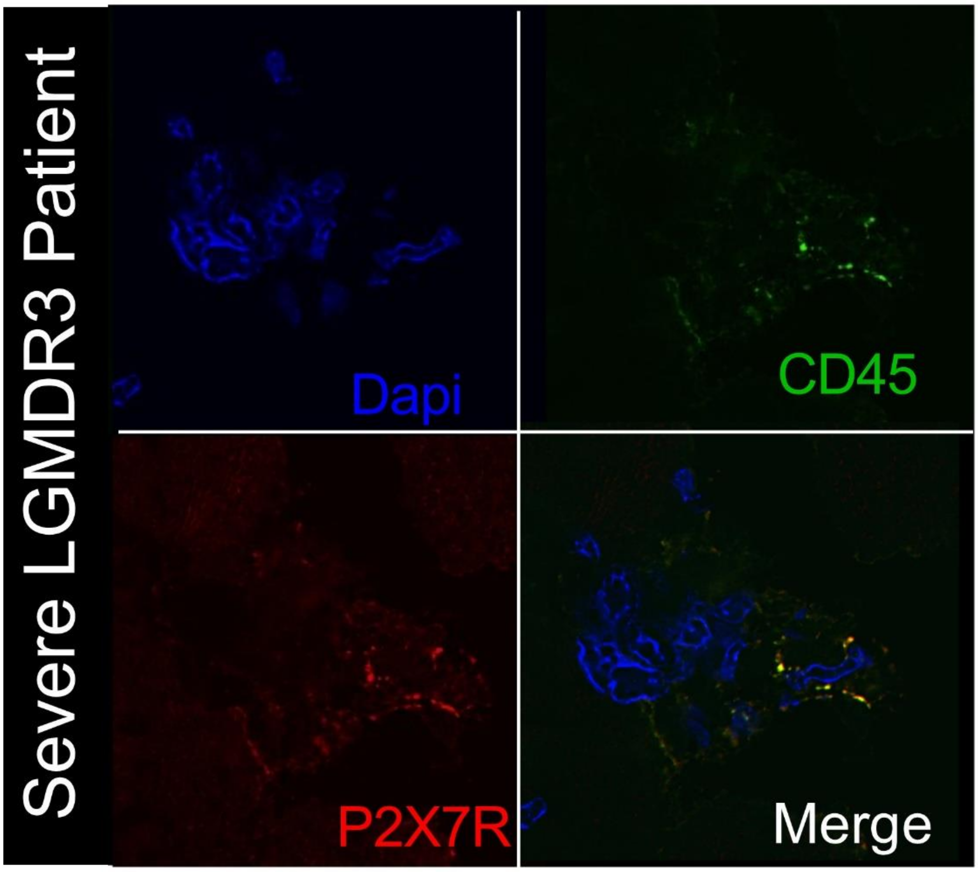

#3

63X magnification of #2 Patient

Supplementary Figure 5.

Evaluation of P2X7R expression in biopsies of skeletal muscle from patients affected by LGMDR3. Enlargement of the images shown in Figure 2, evaluating P2X7R expression in biopsies isolated from healthy subjects (A) and three patients affected by a severe form of LGMDR3 (B). 40X Magnification.

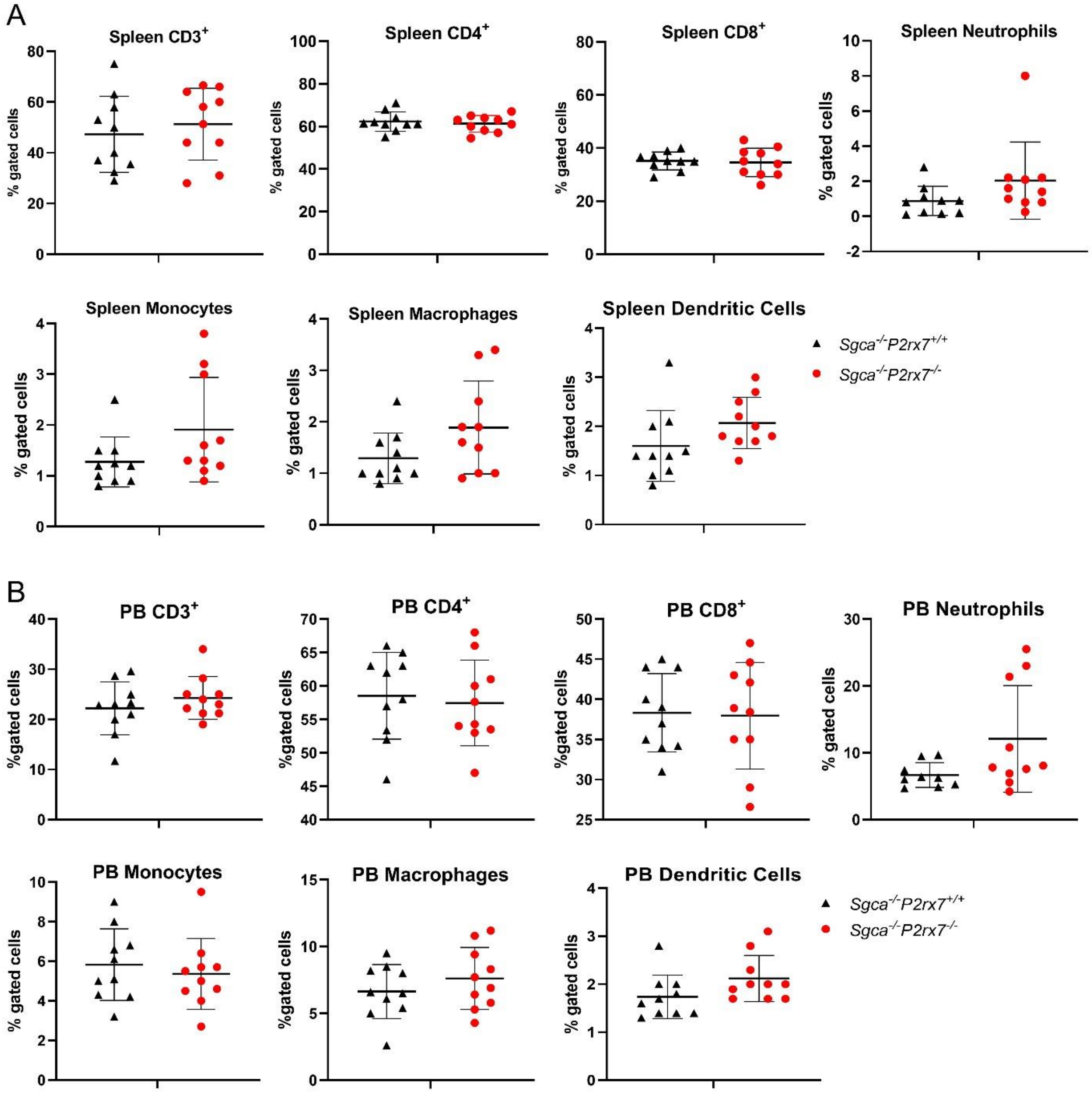

Supplementary Figure 6

Evaluation of inflammatory cells in spleen and peripheral blood. Flow cytometric analysis of immune cells isolated from: (A) spleen and (B) peripheral blood (PB) isolated from muscle tissue of *Sgca$^{-/-}$P2rx7$^{+/+}$* (*n* = 10) and *Sgca$^{-/-}$P2rx7$^{-/-}$* (*n* = 10) mice. Immune cells were stained with specific anti-surface markers: Ly6G, CD11b, F480, CD11c, CD3, CD4, CD8, CD25 and Foxp3.

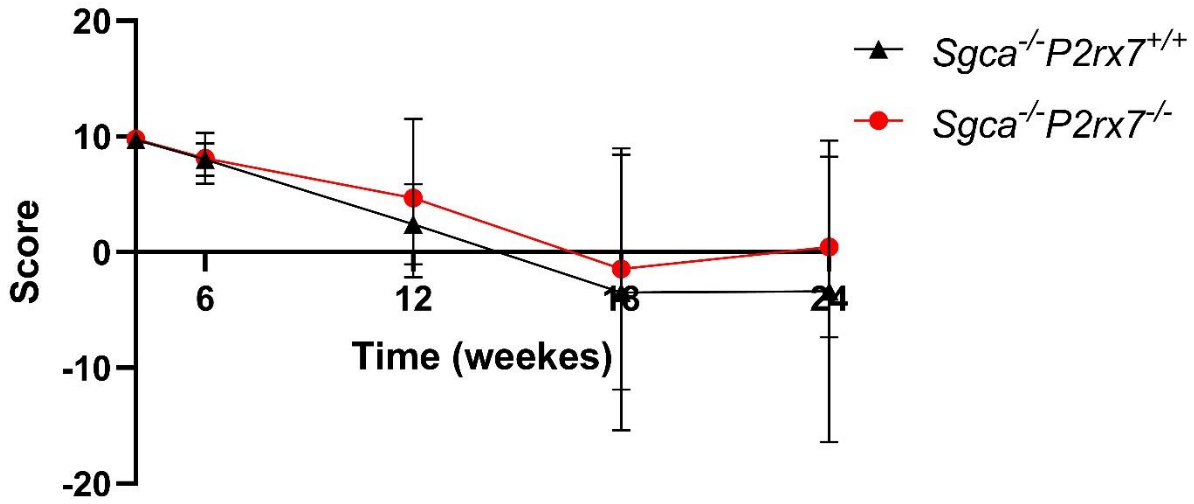

Supplementary Figure 7

Evaluation of motor performance. Four-limb hanging test was performed of 6, 12, 18 and 24 weeks of age. Each value represents the mean ± SD of animals evaluated (*n*=10 for each genotype).

A *Supplementary Figure 8A Sgca*$^{-/-}$*P2rx7*$^{+/+}$*mice*

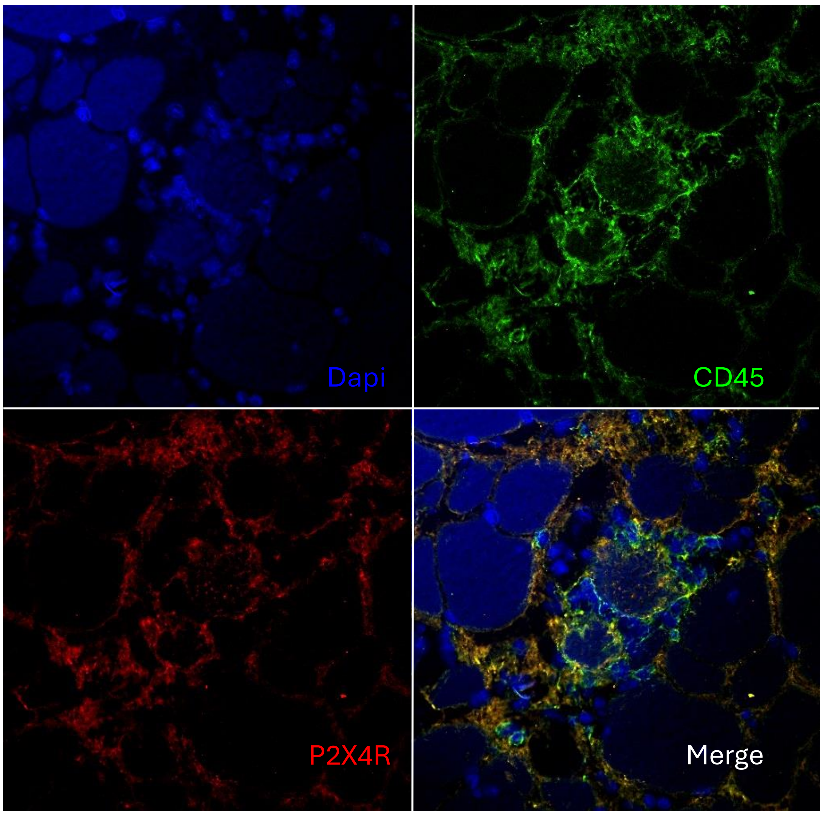

#1 mouse

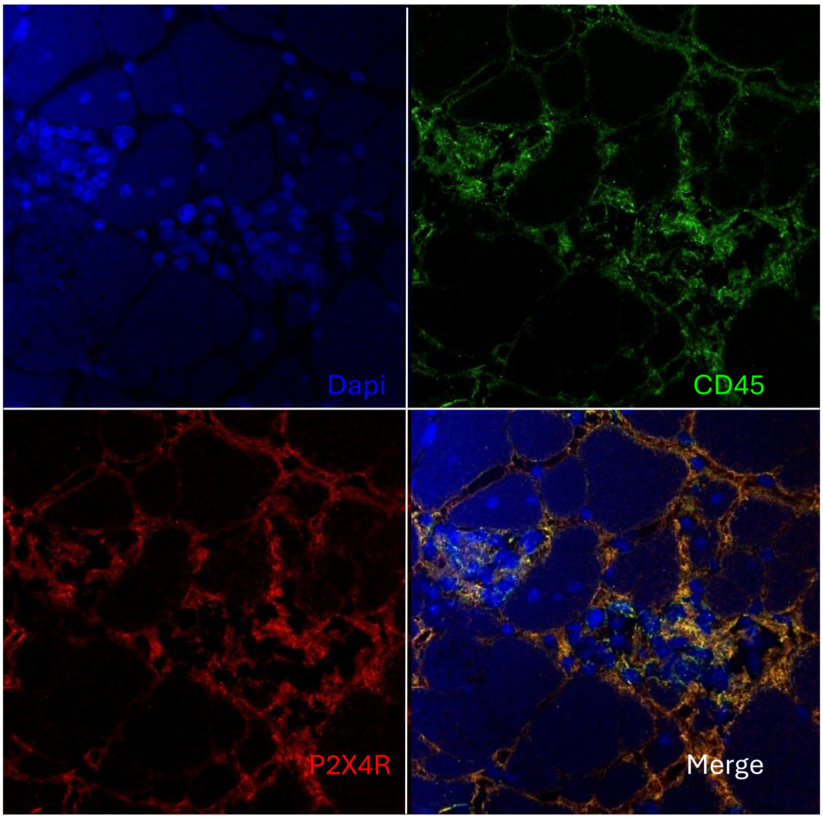

#2 mouse

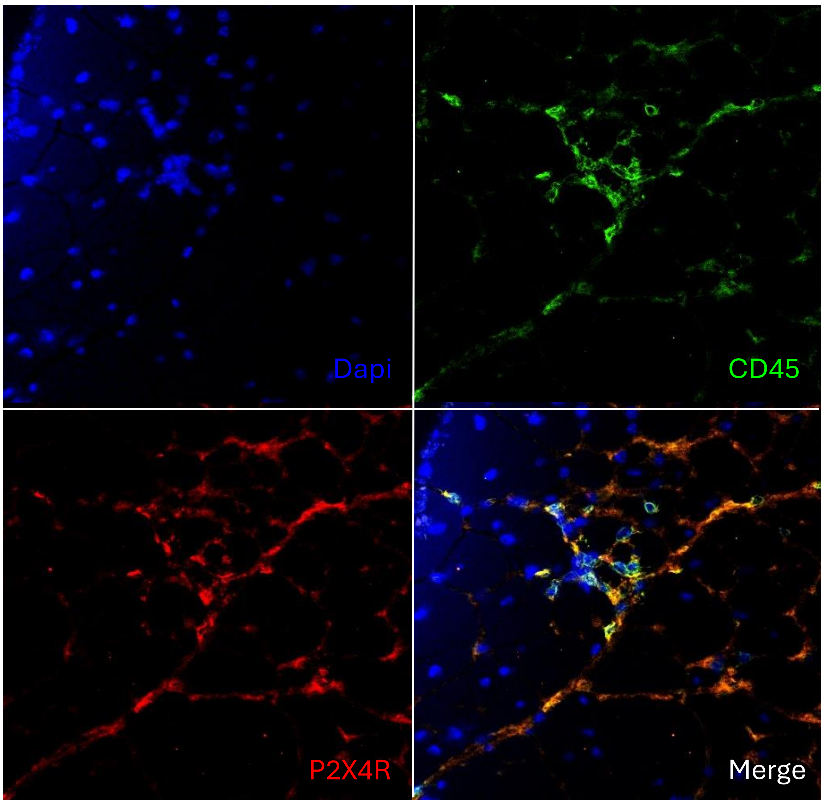

#3 mouse

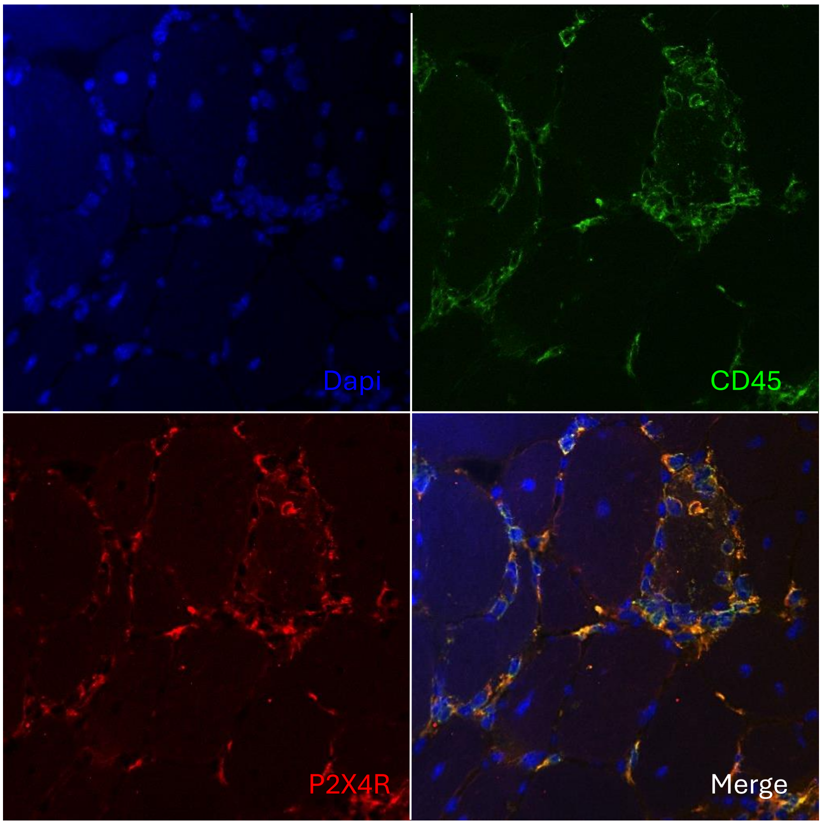

#4 mouse

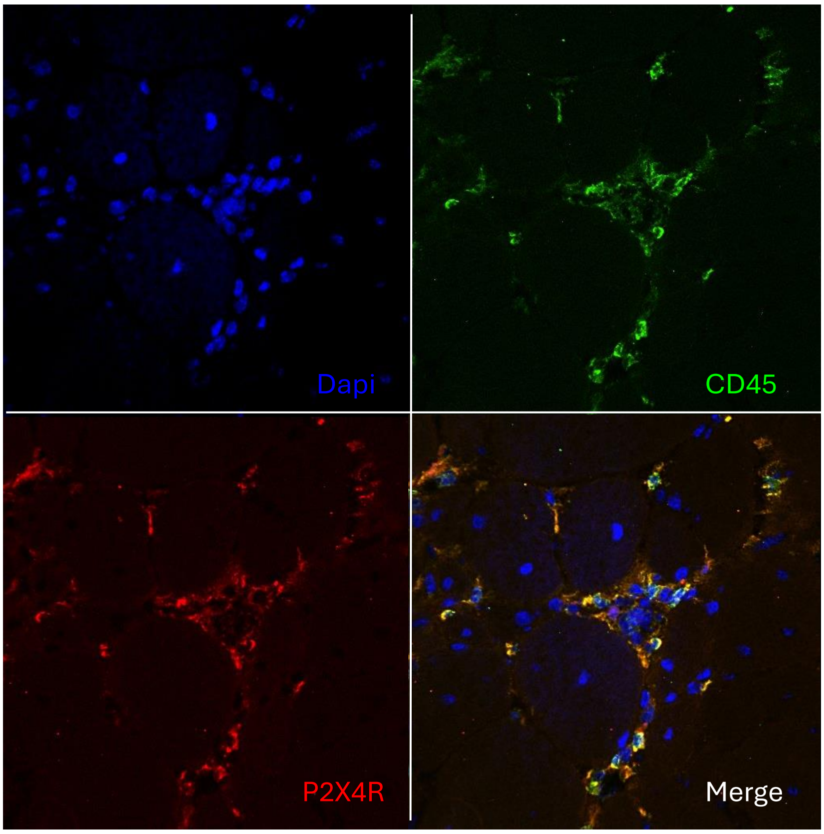

#5 mouse

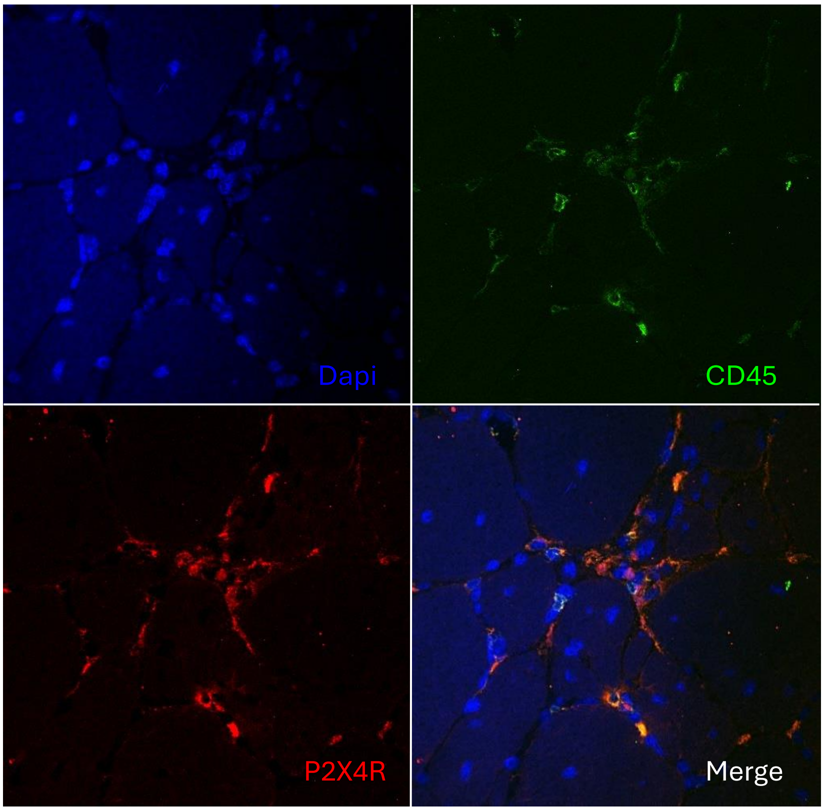

#6 mouse

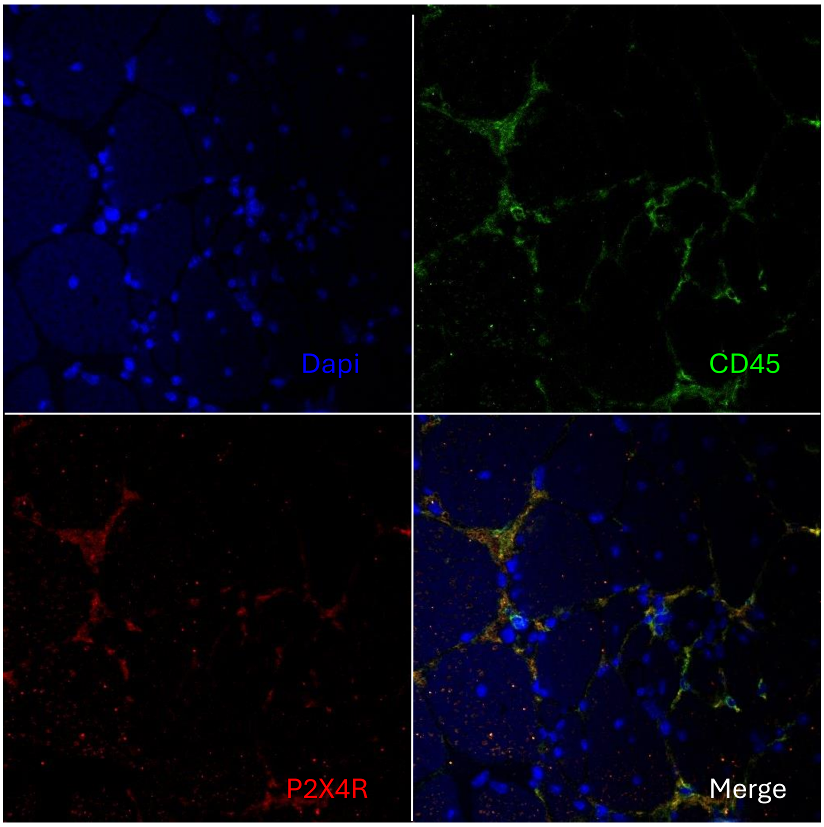

#7 mouse

B *Supplementary Figure 8B Sgca$^{-/-}$P2rx7$^{-/-}$*

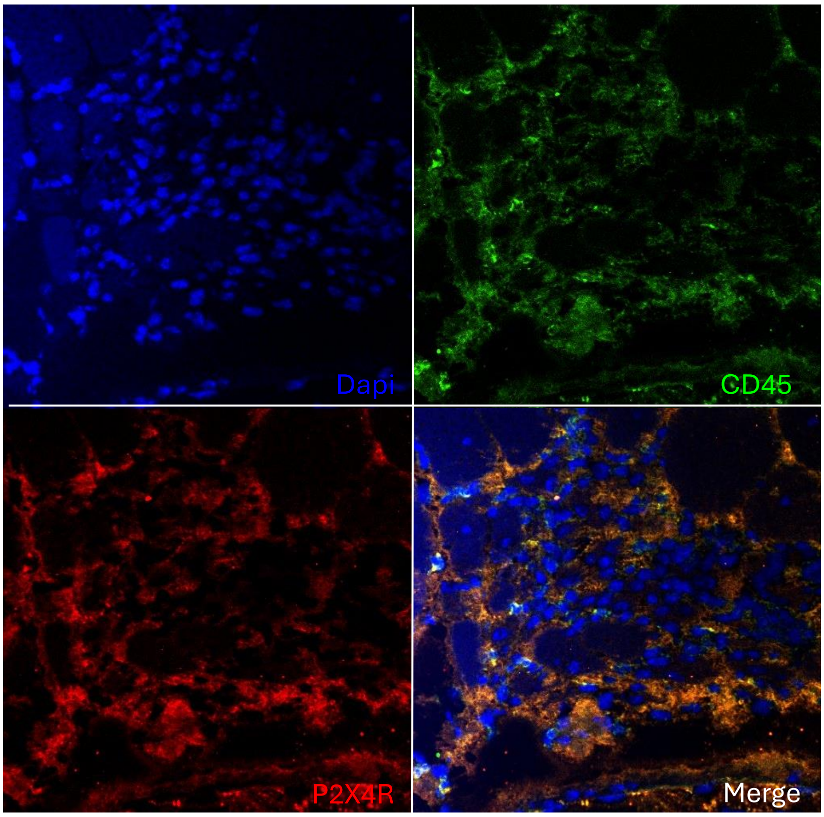

#1 mouse

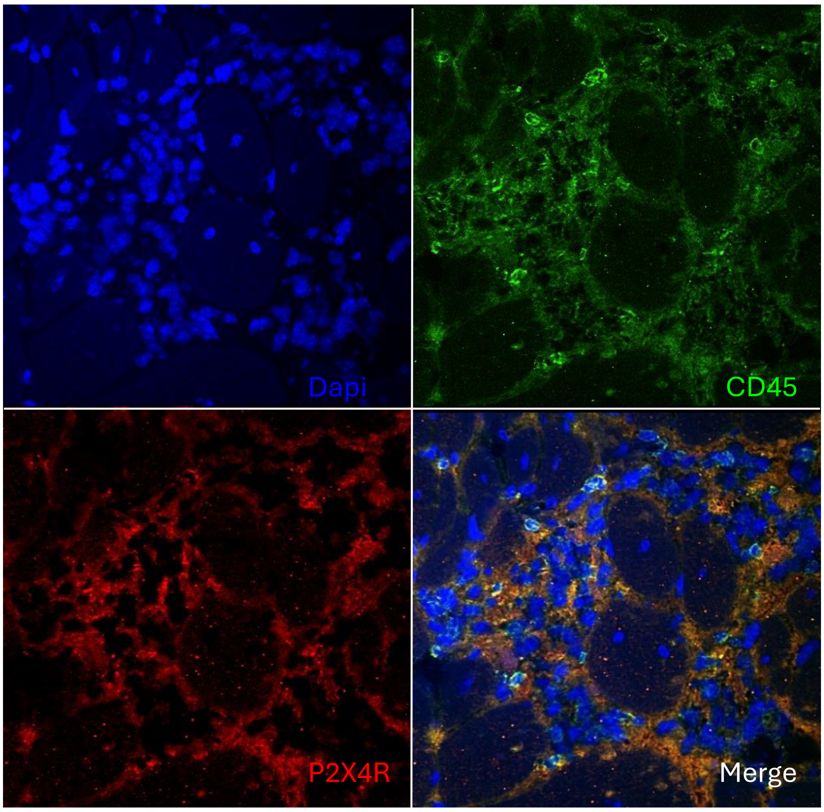

#2 mouse

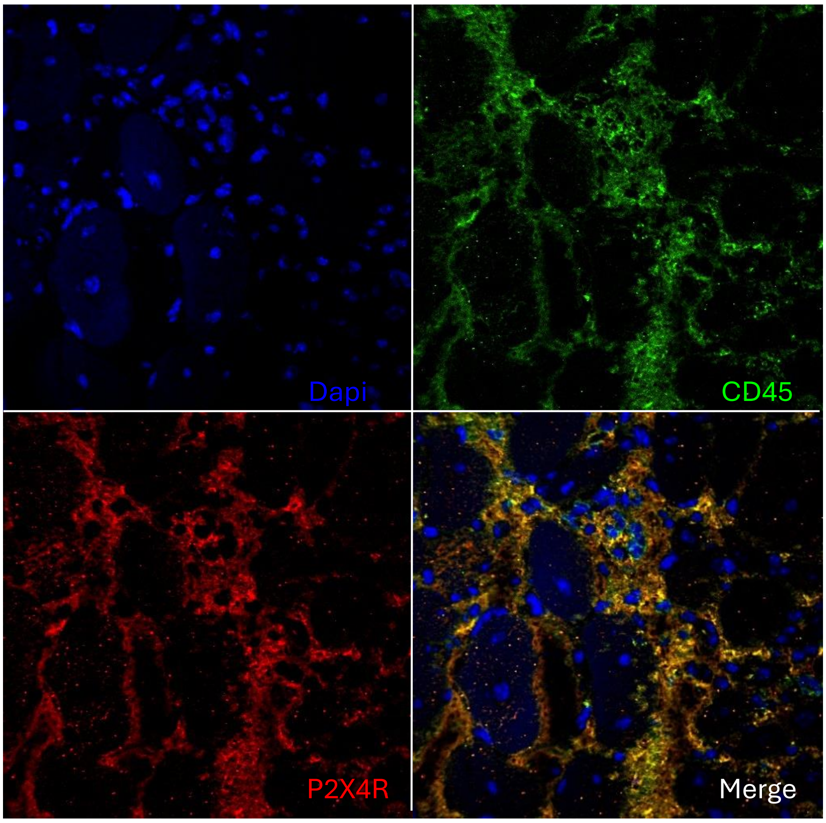

#3 mouse

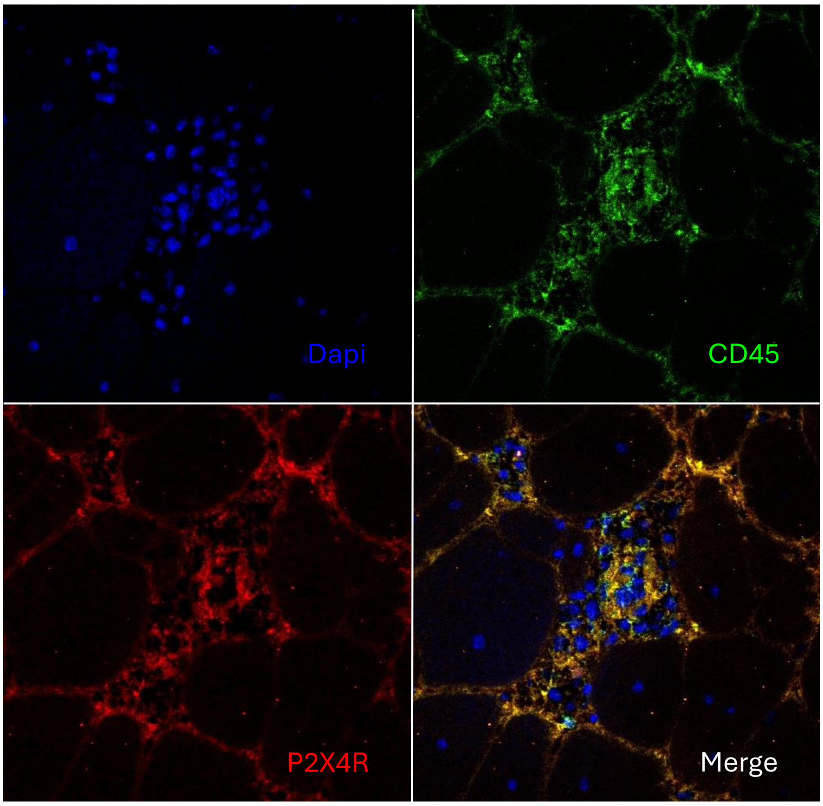

#4 mouse

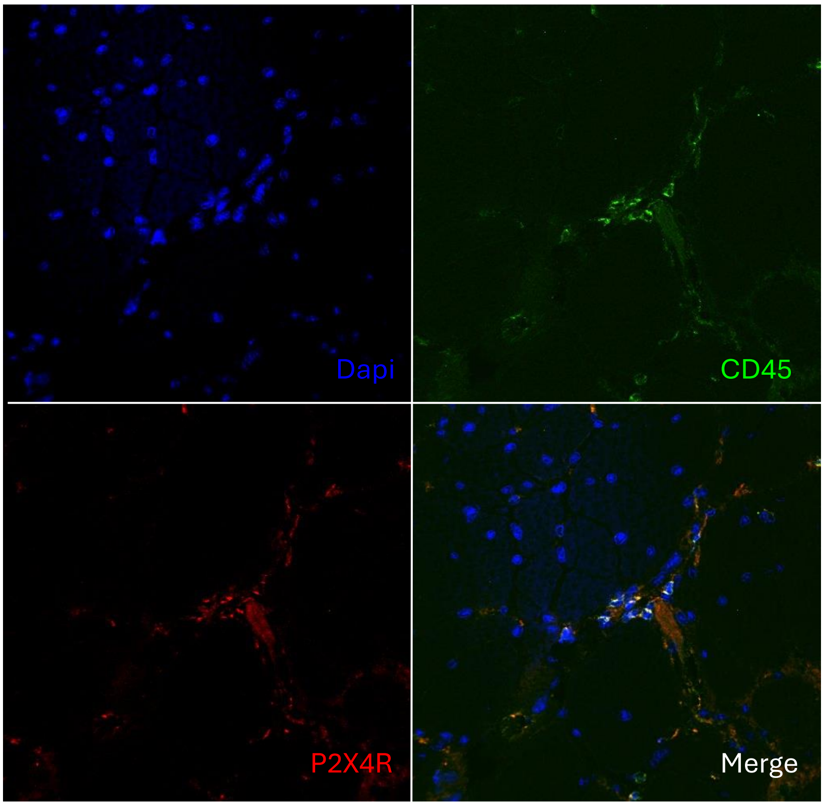

#5 mouse

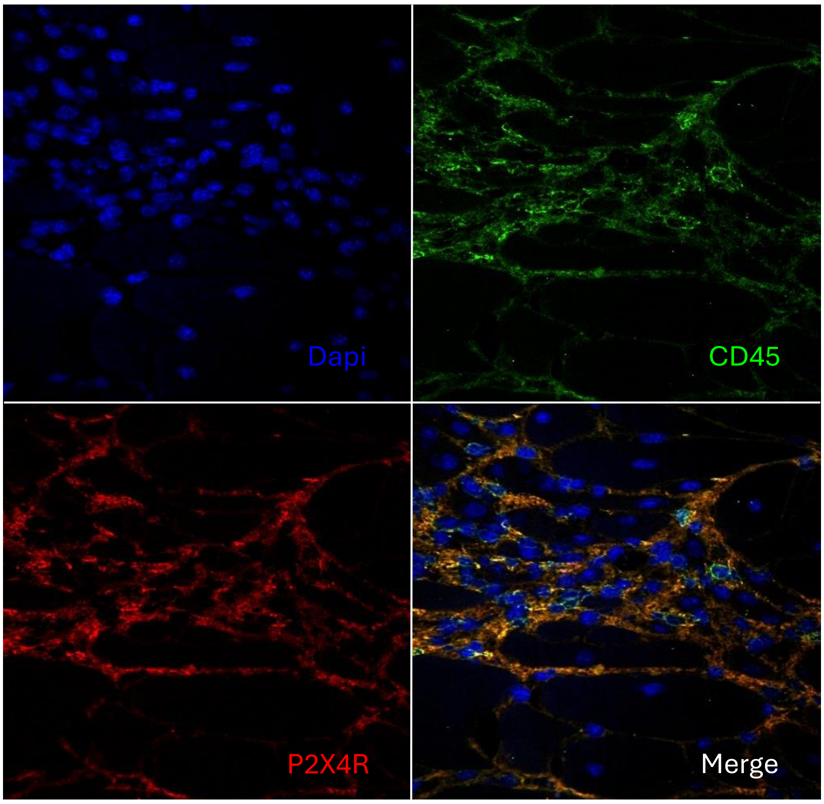

#6 mouse

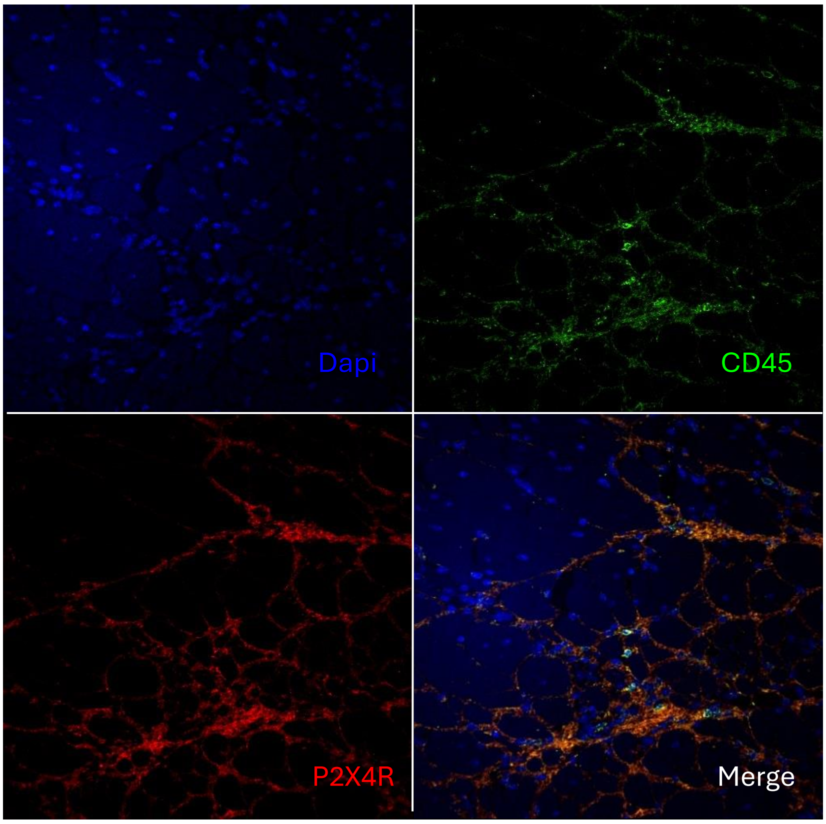

#7 mouse

C

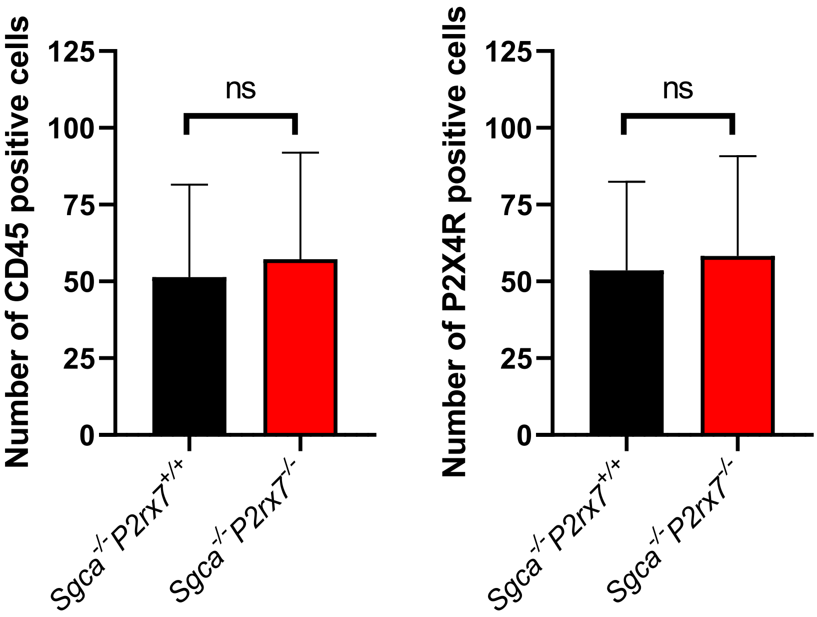

Supplementary Figure 8

Evaluation and quantification of P2X4R and CD45 expression in biopsies of dystrophic muscles of Sgca$^{-/-}$ mice. Representative images of immunofluorescence staining to localize P2X4R and CD45 in skeletal muscle (quadriceps) from *Sgca*$^{-/-}$*P2rx7*$^{+/+}$ (A) *Sgca*$^{-/-}$*P2rx7*$^{-/-}$ (B); 40X Magnification. (C) Quantification of the number of $CD45^{+}$ or $P2X4R^{+}$ cells. *n*=2 images were acquired from 2 slices obtained from *n*=7 animals for each genotype. ns, not statistically different.

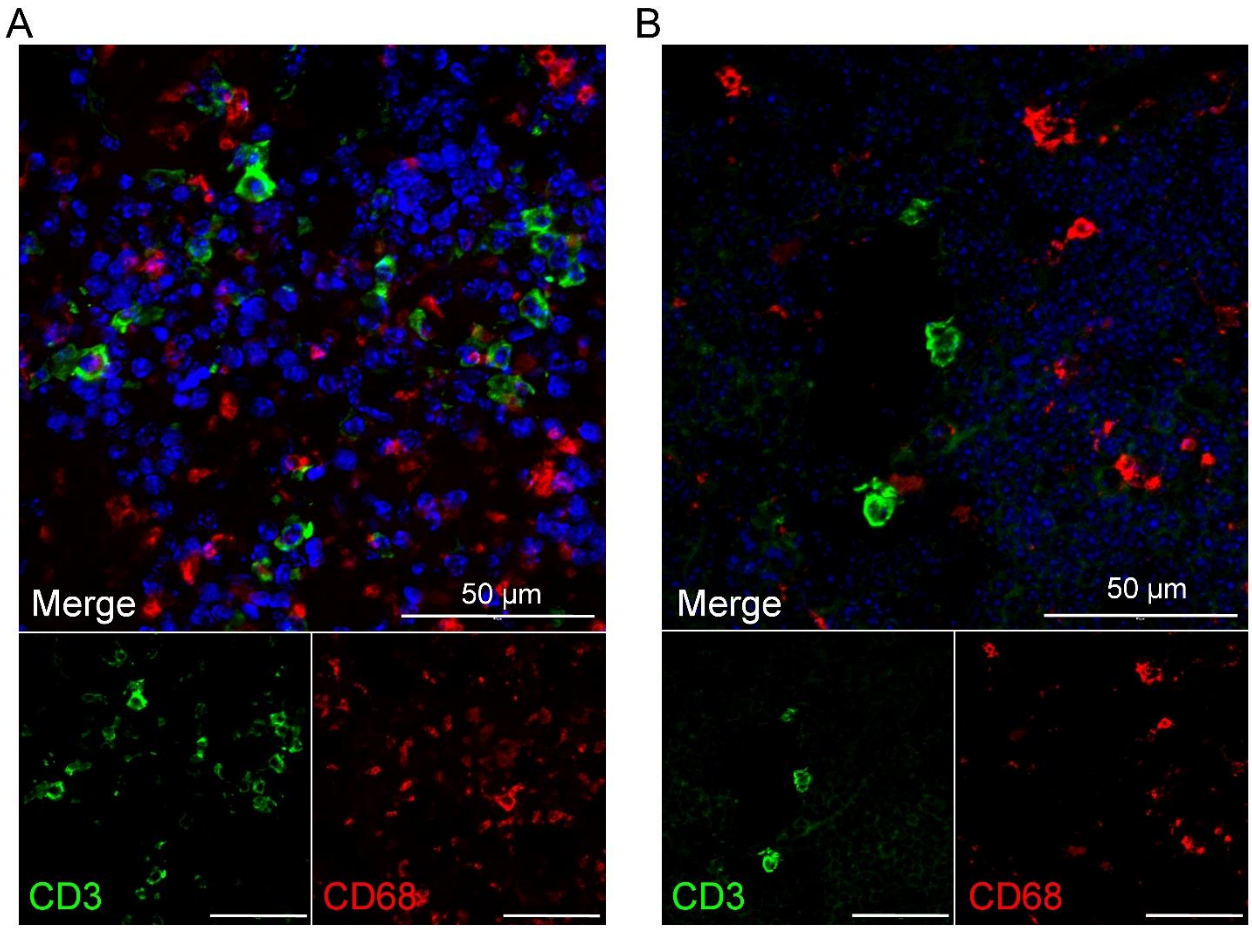

Supplementary Figure 9

Evaluation of CD3 expression in biopsies of spleen and thymus of wild type mice. Representative images of immunofluorescence staining to visualize CD3 and CD68 in spleen (A) and thymus (B) from wild type mice. *n*=3 images were acquired from *n*=2 animals.

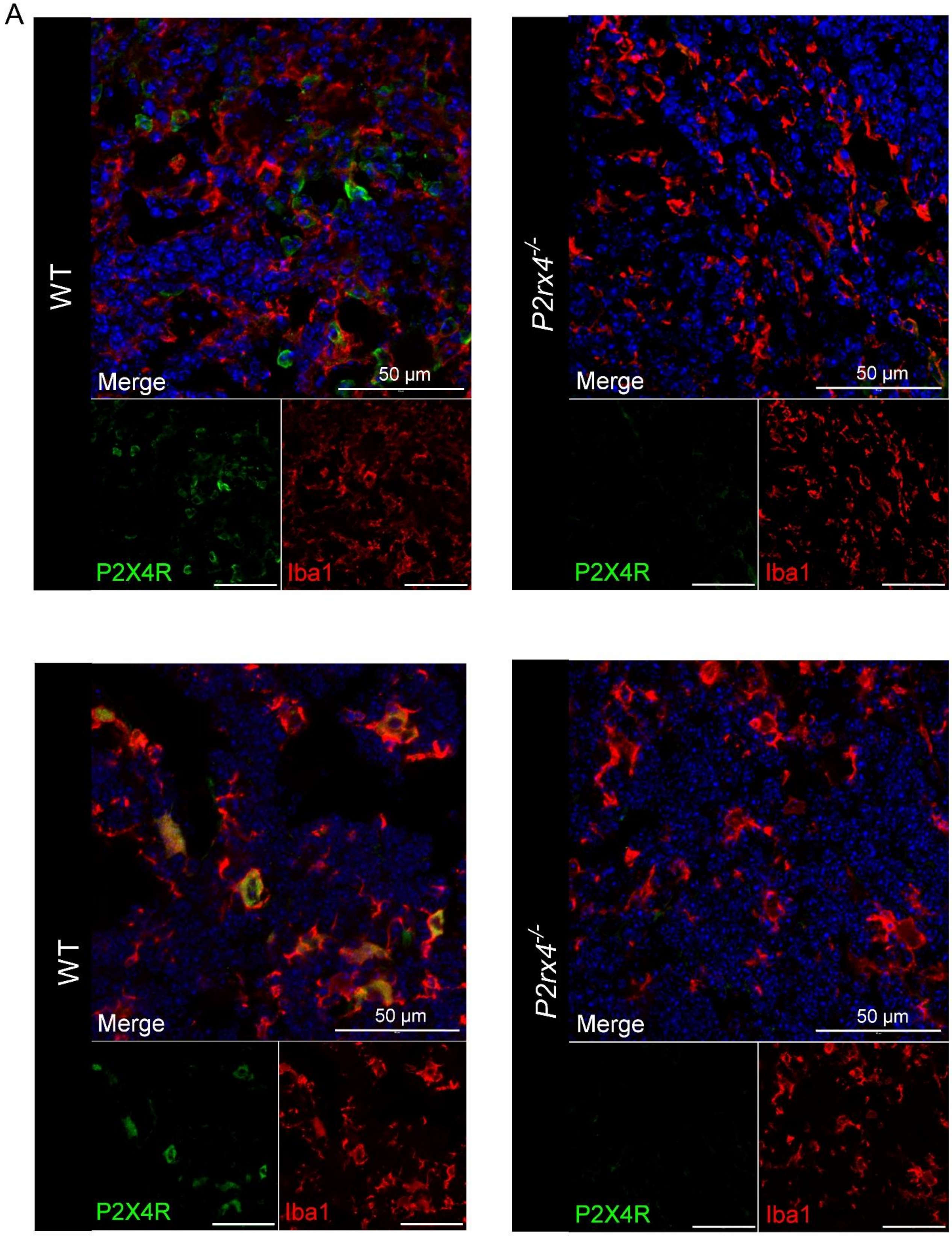

Supplementary Figure 10

Evaluation of P2X4R expression in biopsies of spleen and thymus of wild type and *P2rx4*$^{-/-}$ mice. Representative images of immunofluorescence staining to localize P2X4R and Iba1 in spleen (A) and thymus (B) from wild type and *P2rx4*$^{-/-}$ mice. n=3 images were acquired from n=2 animals.
